# Technological, healthcare and consumer funds efficiency: influence of COVID-19

**DOI:** 10.1007/s12351-023-00749-x

**Published:** 2023-04-05

**Authors:** Catarina Alexandra Neves Proença, Maria Elisabete Duarte Neves, Maria do Castelo Baptista Gouveia, Mara Teresa da Silva Madaleno

**Affiliations:** 1 University of Coimbra, CeBER, Faculty of Economics, Coimbra, Portugal; 2 Polytechnic Institute of Coimbra, Coimbra Business School | ISCAC, Coimbra, Portugal; 3 Universidade de Trás-os-Montes e Alto Douro| CETRAD, Vila Real, Portugal; 4 INESC Coimbra - DEEC University of Coimbra , Polo 2, 3030-290 Coimbra, Portugal; 5 GOVCOPP–Research Unit in Governance, Competitiveness and Public Policy, Department of Economics, Management, Industrial Engineering and Tourism (DEGEIT), University of Aveiro, Aveiro, Portugal

**Keywords:** Investment funds, Efficiency, COVID-19, Healthcare, Technology, Consumer cyclical, DEA, PCA

## Abstract

This paper aims to analyze the efficiency of the funds in technological, healthcare, and consumer cyclical sectors based on the U.S. News & World Report rankings. We employed a Principal Component Analysis to select the indicators to explain efficiency. Then, we have used an alternative approach that combines Data Envelopment Analysis (DEA) with Multiple Criteria Decision Aiding, the Value-Based DEA, to assess the efficiency of funds for 1 year (2020), 3 years (2018–2020), and 5 years (2016–2020). The results highlight that in 2020 the number of efficient funds is much smaller than in previous periods and this can be justified by the effect of the COVID-19 pandemic crisis. The sectors with the most efficient funds are technology and healthcare. The factors that determine the efficiency of funds in the health sector and the technology sector are quite different, although they have not undergone major changes in the three periods considered. For managers, health funds are seen as low risk and hardly consider the return factors in all analyzed periods, which is often considered as benchmarks for inefficient funds. In the technology sector, Beta and Alpha are generally the indicators with the greatest weight in fund efficiency, showing that these funds beat the market in terms of returns and are less risky than the benchmark. This study seeks to complete the scarce existing literature on the subject, namely in the sectors under analysis, seeking to identify the indicators that fund managers ponder most to consider a fund as efficient. As far as we know, the joint efficiency analysis of these sectors and the impact they suffered from the COVID-19 pandemic are new in the literature.

## Introduction

The COVID-19 outbreak was similar to a global economic crisis (Hasnaoui et al. 2021). There was an increase in volatility in global markets (Shehzad et al. 2020; Zhang et al. 2020), a change in stock performance (Corbet et al. 2020), an increase in petrol prices, and increased geopolitical risk (Sharif et al. 2020), a shift to gold and cryptocurrencies investments (Corbet et al. 2020; Mnif et al. 2020). Particularly for financial markets, the uncertainty inherent in COVID-19 required financial market participants to adjust. Yarovaya et al. (2020) concluded that the response given by the stock market, bonds, precious metals, and cryptocurrencies was different, as its recovery. Concerning investment funds, there was also a change in their performance.

For example, Rizvi et al. (2020) concluded that to respond to the challenges of the pandemic, fund managers changed their investment style (Hasnaoui et al. 2021; Yarovaya et al. 2020). By this time, many funds have outperformed the passive benchmarks (Hasnaoui et al. 2021). The literature also shows that funds facilitate periods of stress because, due to their active investment strategies, fund managers can produce consistent positive Alphas (Huang et al. 2021).

Moreover, the COVID-19 lockdowns emphasized the importance of three specific sectors—health, technology, and consumption. For example, at this time, the world observed the inability of hospitals to effectively care for all patients; the lengthy period to develop the vaccine that led to the disastrous isolation problems of the citizens; the creation of teleconsultations that prevented many users from being treated for other chronic diseases, the advantages and disadvantages of online education and the increase in e-commerce (e.g., Elrhim and Elsayed 2020; Iyengar et al. 2020; Shenoy et al. 2020). Moreover, the new normal provoked by COVID-19 implied changes in telecommunications, new technologies, and information technology companies (Ntasis et al. 2021).

Thus, investment funds related to these three sectors could be gain investors’ interest. Indeed, the healthcare sector has grown given the aging population, and in common sense, investing in this sector can be more profitable than investing in an index, as this sector brings benefits to the public (Chen et al. 2018). For these authors, the healthcare sector is defensive against market rebounds and is actively managed, investing in healthcare-related companies (e.g., hospitals, pharmaceuticals, medical equipment industry). Investors can keep health funds in their portfolios as a hedge against the risk of a market downturn (Chen et al. 2018). Another study, Martí-Ballester (2020b) shows that biotechnology and healthcare funds can outperform conventional funds due to the ability of managers to choose stocks that are undervalued in the markets. Moreover, Martí-Ballester (2020a) shows that pension funds that invest in healthcare and technology can beat the market. The technology sector is crucial for survival in a competitive world (Sohn et al. 2007). However, according to Maruti and Shivaji (2013) technology funds are not suitable for conservative investors, noting that none of these funds outperformed their benchmarked portfolios. Boulatoff and Boyer (2017) conclude the opposite, noting that cleantech funds performed better than the benchmark. Regarding the consumer cyclical sector, this represents goods and services considered as luxury and not as a first necessity, being related to the state of the economy. Thus, in periods of crisis, investors will have less disposable income to invest in this sector (Gejalakshmi and Azhagaiah 2017). Meric et al. (2010) conclude that in 2009 US consumer and healthcare funds had lower Betas and better performances compared to technology.

Investors use various indicators to assess the fund’s performance and efficiency and choose the funds in which to invest. Sirri and Tufano (1998) shows that consumers base their fund purchase decisions on prior performance information, so performance measures are widely used by these. However, investors are interesting too in management risk (Simons 2000). More recently, evaluating the fund’s efficiency becomes a very important topic for analysis and popular among investors through diversification and competitiveness returns (Walia and Kumar 2013). In addition, COVID-19 is also an opportunity to analyze whether the efficiency of funds in three sectors has changed, allowing comparative studies in the future.

This paper aims to assess the characteristics that most contribute to the investment funds efficiency in sectors of technology (44 funds), healthcare (30 funds), and consumer cyclical (7 funds) for 1 year (2020), 3 years (2018–2020) and 5 years (2016–2020), with a particular focus on the year 2020 with the effects of COVID-19.

However, the literature shows the undifferentiated use of inputs and outputs to measure efficiency. In this sense, and to fill this gap, we employ a Principal Components Analysis (PCA) to determine the main determinants for efficiency, namely the inputs and outputs. Then, we have used a method based on Data Envelopment Analysis (DEA) which is a mathematical programming technique that produces an efficiency frontier by comparison of homogeneous decision-making units (DMUs), considering multiple inputs and multiple outputs.

This work aims to expand the existing empirical literature, analyzing the efficiency of three sectors considered the most affected by the COVID-19 pandemic crisis. Thus, we intend to answer three important research questions: i. What was the evolution of the efficiency of funds in the sectors most affected by COVID-19 in the period from 2016 to 2020; ii. What are the factors that determine the efficiency of these funds, are they return measures, risk and/or risk-adjusted return measures? and, iii. Do the factors vary from industry to industry?

As far as we know, this study has not been done before. At the same time, this study aims to assist managers and investors in their investment decision-making. The main motivation for carrying out this research is related to the fact that these funds are increasingly an investment alternative, and it is necessary to demystify the indicators that matter when making an investment decision.

The remainder of the paper is organized as follows. Section 2 surveys the relevant literature on investment fund's performance. Section 3 describes the data and methodological framework. The results for the dynamic evaluation are presented in Sect. 4 and Sect. 5 provides some conclusions, limitations, and lines for future research.

## Literature review

Investment funds are a financial instrument that results from raising capital from several investors, forming the set of these amounts as an autonomous asset, managed by specialists who invest it in a diversity of assets that allow diversification (Neves et al. 2019). Jensen (1968) and Sharpe (1966) carried out the classical research for investment funds performance. Jensen (1968) proposed a widely used model to assess the funds’ performance in the scientific world. This model was based on the Alpha measure, a fund’s performance indicator compared to its benchmark, and includes the Capital Asset Pricing Model (CAPM) developed by Sharpe (1964) in collaboration with Lintner (1965) and Mossin (1966). Over time, new models and more studies have arisen to analyze the funds’ performance evolution, such as the multifactorial model by Fama and French (1993). Recently there have been multiple studies with controversial conclusions about the determinants of the efficiency and performance of funds.

Traditionally, the funds’ performance was evaluated by returns and risk factors, such as Treynor ratios (1965); Sharpe index (1966), or the Treynor and Mazuy’s model (1966), as well as the Carhart (1997). Indeed, recently, a greater number of studies have considered other characteristics as potential determinants of funds’ performance. Some of these attributes explaining mutual funds portfolios’ performance include past returns (Ippolito 1989); size (Grinblatt and Titman 1989; Yan 2008); liquidity (Amihud et al. 2005; Chen et al. 2004; Schaub and Schmid 2013); age (Pástor et al. 2015); fees or global costs (Wermers 2000); incentive fees (Edwards and Caglayan 2001); Standard deviation or gross returns (Gouveia et al. 2018; Kenchington et al. 2019; Henriques et al. 2022); among others.

The DEA methodology was originally proposed to evaluate the performance of production units (DMUs), where the efficient frontier DEA can be considered as an empirically derived production frontier.

The seminal work by Murthi et al. (1997) was the starting point for the application of the DEA methodology in the evaluation of investment funds. This work proposes a DEA portfolio efficiency index (DPEI). Since this first work, the DEA has been widely used to evaluate the performance of funds (Singla and Gupta 2020).

In recent years, DEA has been used for performance evaluation and benchmarking against best practices, i.e., it has been seen as a Multi-Criteria Decision Aid (MCDA) tool. In this work, the Value-Based DEA is used, which combines DEA with MCDA and therefore as a multicriteria decision support tool, in which “*the inputs are usually the “less-the-better” type of performance measures and the outputs are usually the “more-the-better” type of performance measures*” (Cook et al. 2014) and where to have knowledge of which is the production process does not make any sense.

In the literature, there are few works that address the performance evaluation of funds with methodologies that link DEA with MCDA. However, there are some that mingle these two methodologies together with the aim of including an investor/portfolio manager's preferences (for a comprehensive review, see Zopounidis et al. 2015).

The present work is in line with the view of Tarnaud and Hervé (2018), who say that this type of approach (DEA combined with MCDA) to value financial assets is not restricted to a risk-return analysis, but can be seen as a cost-effective approach to investor preferences. Therefore, from an investor's perspective, the factors being evaluated should be those considered relevant by a typical investor who wants to evaluate the performance of his portfolio. Still following the perspective of these authors and assuming the integration of risks as undesirable characteristics of funds, it is assumed that investors' preferences for risks are restricted to risk aversion or mixed risk aversion.

In this literatute, it makes sense that the outputs (factors to be maximized) are defined as the benefits obtained by the investor when carrying out the investment, such as the gross return, and the inputs (factors to be minimized) are defined as the resources spent by the investor (loads, such as sales charges, redemption fees and other fund expenses) and the various measures of risk, such as the standard deviation or beta.

It is also verified that there is an incidence of studies in the analysis of risk and return measures, as the investor will always have the consideration of the risk-return binomial in his analysis. Thus, the main measures that have been used in these two areas are highlighted below and resumed in Table 1. We emphasize that this table represents the most relevant studies that focus on the subject, with the presentation of some seminal investigations and with a strong focus on studies after 2015. Basso and Funari (2016b) present the literature review from 1997 to 2015, so this is not repeated in this study.

**Table 1:** Studies on DEA in the performance evaluation of mutual funds with a strong focus after 2015

Authors	Inputs	Outputs	Data
Murthi et al. (1997)	Expense ratio, load, turnover, standard deviation	Return	Mutual funds 1993
Chang (2004)	Standard deviation, Beta, Assets	Returns	US mutual funds 1992–1996
Chen et al. (2011)	Monthly purchasing turnover rate, Direct transaction cost rate, Selling expense rate, Monthly standard deviation	Treynor index, Sharpe index, Jensen index, Monthly return rate	Mutual funds 2007
Matallín-Sáez et al. (2014)	Standard deviation, Kurtosis daily returns, Expense ratio, Beta	Gross Return, Skewness daily returns	US mutual funds 2001–2011
Tsolas and Charles (2015)	Portfolio Price/Cashflows Portfolio Price/Book	Sharpe index, Jensen’s alpha	Green ETF 2008–2010
Basso and Funari (2016a)	Payout required to an investor by fund, Beta	Final value of the investment	European equity mutual funds 2006–2009
Basso and Funari (2016b)	Payout required to an investor by fund, Standard Deviation, Beta, Downsize risk	Final value of the investment	European mutual funds 2006–2013
Premachandra et al. (2016)	Management fees, Marketing and distributions fees, Fund Size, Net expense ratio, Turnover, Standard Deviation, Adjusted net asset value	Net Asset value, Return	US mutual funds 1993–2008
Gardijan and Krišto (2017)	Semi-variance, Expected Shortfall	Excess return, skewness, Standard deviation	Croatian Mutual funds 2005–2015
Lin et al. (2017)	Conditional Value at Risk, Loss risk of fund, Expense ratio, Management fees	Return	Chinese and European mutual funds 2004–2013
Basso and Funari (2018)	Payout, Beta, Downside risk	Final value	European mutual funds 2006–2013
Galagedera (2018)	Turnover, Management fee, Fund size, Net Asset Value, Total risk, Downside risk, Systematic risk	Morningstar portfolio ESG score, Benefit payments, Net Assets Value, Return	US mutual funds 2016
Gouveia et al. (2018)	The proportion of negative monthly returns during the year, Expense ratio, Standard deviation, Beta	Gross return	Portuguese equity funds 2007–2014
Vidal-García et al. (2018)	Expense ratio, Turnover, Load, Standard deviation	Return	Worldwide equity funds 1990–2015
Allevi et al. (2019)	Beta, Initial payout invested, Downside risk, Environmental consumption indicators	Final value, Environmental saving indicators, Green indicator	European green funds 2010–2015
Lin and Li (2019)	CVaR deviation Expense ratio Holdings turnover	Expected return	US mutual funds 2017
Tsolas (2020)	Management expenses, Standard Deviation, Front load plus deferred load	Net asset value, Return	Precious metal mutual funds 2005–2015
Tuzcu and Ertugay (2020)	The expense ratio, Management fees, Capital	Net asset value, Total Assets, Total market value	Turkish mutual fund market 2005–2007
Walavalkar et al. (2020)	The expense ratio, Asset under management, Standard deviation, Sharpe ratio, Sortino ratio, Beta, Alpha, R-Squared	Return	Indian mutual funds 2015–2020
Bilbao-Terol et al. (2021)	Conditional Value at Risk	Return	French Mutual Funds 2006–2015
Tian et al. (2022)	Variance, Liquidity risk	Return	Chinese short-term mutual funds
Henriques et al. (2022)	Standard deviation, Beta	Jensen’s Alpha, Sharpe index, mean annual return, trailing total return	ETF in the energy sector 2009–2019
Shahrour (2022)	Standard deviation, Beta, Value at risk, Conditional Value at Risk, Net asset value, Expense ratio	Capitalization factor, nvironmental, Social and Corporate Governance (ESG) rating	French socially responsible mutual funds

As we can see, there are several measures to assess mutual fund performance. We present below a brief definition and explanation of the variables that have been most used as risk and return indicators, which are even made available in funds’ databases.

### Risk measures

#### Standard deviation

The Standard deviation was used in several studies (e.g., Chang 2004; Chen et al. 2011; Gouveia et al. 2018; Tsolas and Charles 2015) to assess the volatility of returns over a certain period, i.e., it represents the total risk of the portfolio (Tuzcu and Ertugay 2020). If this value is close to zero, it means that the data are more uniform, that is, the sampling presents return values that are closer to each other. On the other hand, the higher its value, the more volatile the fund will be. Thus, this indicator is normally used by investors to predict the volatility of funds. The Standard deviation corresponds to the square root of the variance. Thus, the Annualized standard deviation of a fund is calculated as follows:

1 $$\sigma_{m} = \sqrt {\frac{{\mathop \sum \nolimits_{i = 1}^{n} \left( {R_{i} - \overline{R}} \right)^{2} }}{n - 1}}$$

where, $\sigma_{m}$ is the Standard deviation of the annual return rate, $R_{i}$ is the annual rate of the *i*th year and $\overline{R}$ is the mean annual return rate. It is also possible to use the variance, which corresponds to the square of the standard deviation.

#### Beta

Beta is another risk indicator traditionally used (e.g., Chang 2004; Huang et al. 2015; Matallín-Sáez et al. 2014). This indicator assesses market risk, or systemic risk, considering portfolio diversification (Lin and Li 2019). This indicator is calculated through the covariance (*Cov*) of the return on an asset (Rj) with the benchmark or market return (Rm), divided by the variance of the benchmark return in a given period, and then given by the following formula:

2 $$\beta_{j} = \frac{{Cov\left( {R_{j} ,R_{m} } \right)}}{{Var\left( {R_{m} } \right)}}$$

If the Beta is greater than one, it means it is more volatile than the benchmark, while a Beta value of less than one means the fund is less volatile than the market and therefore less risky.

#### Correlation and R-squared

The R-Square was also analyzed as an explanatory factor for efficiency (Guo 2015; Walavalkar et al. 2020). The R-Square represents the percentage of a fund’s portfolio that can be explained by movements in a reference index, i.e., it is the relationship between a portfolio and its benchmark. It is given by the following relationship:

3 $$R - Square \, = \, Correlation^{2} = \left[ {\frac{{Cov\left( {Benchmark, Fund} \right)}}{{\sigma_{Fund} *\sigma_{Benchmark} }}} \right]^{2}$$

where $\sigma$ is the Standard deviation.

#### Tracking error

The Tracking error has been used by Rizvi et al. (2020) and Tsolas and Charles (2015). These authors show that the Tracking error measures relative risk as it indicates the degree to which a portfolio approaches or does not approach its benchmark. This measure is then given by the standard deviation of the difference between the portfolio return and the benchmark return:

4 $$Tracking error = \sigma \left\{ {R_{p} - R_{M} } \right\}$$

where *Rp* is the return of the portfolio; *R*_*M*_ the return of benchmark.

This measure is more important for investors, who at least want to obtain returns close to the benchmark, than for managers, whose objective is to beat the market (Gastineau 2010).

#### Value-at-risk

Branda (2016) and Khanjani and Madjid (2020) use the value-at-risk measure to express risk. This measure shows the loss potential associated with an investment over a given period. This measure outperforms the Markowitz mean–variance method (Khanjani and Madjid 2020), being a popular measure for calculating risk (Branda 2016). Calculating this indicator is through parametric methods, where it is necessary to know the normal curve, and non-parametric methods, where historical or Monte Carlo simulations are used.

#### Maximum drawdown

The maximum drawdown explains the largest percentage drop from peak to trough before a new peak in a specific mutual fund during a specific period (Sharma and Sharma 2018). This indicator captures how well the mutual fund can rebound (Gregoriou 2006). In Hassan and Merdad (2012) study, this indicator was estimated as the maximum number of months that a specific fund has been below a historically high net asset value.

### Return measures

#### Alpha

Jensen’s alpha has also been used in the literature by authors such as Chen et al. (2011), Tsolas and Charles (2015), or more recently by Henriques et al. (2022). Alpha is a risk-adjusted measure that measures a fund’s excess return above or below the CAPM forecast. This indicator measures which rate of return is capable of providing above-average returns, adjusted for market risk. Alpha with a positive value indicates better risk-adjusted returns, that is, it means that the fund can outperform its benchmark, if we find a negative Alpha, it means that the manager cannot add value to the fund and indicates worse returns than the market. Alpha could be expressed as the following equation:

5 $$\alpha_{i} = \overline{{R_{i} }} - \left[ {\overline{{R_{f} }} + \beta_{i} \left( {\overline{{R_{m} }} - \overline{{R_{f} }} } \right)} \right]$$

where $\alpha_{i}$ is the Jensen’s alpha for the ith mutual fund, $R_{i}$ is the annual mean return rate for the ith fund, $\overline{{R_{f} }}$ is the riskless return rate, $\overline{{R_{m} }}$ is the mean return rate of the market portfolio and the $\beta_{i}$ is the systematic risk for the ith mutual fund as expressed in subSect. 2.1.1.

#### Sharpe ratio

Sharpe (1966) has developed a measure, the Sharpe ratio, to assess the relationship between risk and return of the funds. It is a classic risk-adjusted-performance indicator, which does not need a benchmark, making it quite pleasant, given the inherent difficulty in choosing the most appropriate benchmark.

6 $$Sharpe Ratio = \frac{{R_{p} - R_{f} }}{{\sigma_{p} }}$$

where *Rp* is the return of the portfolio; *Rf* the risk-free rate and $\sigma_{p}$ is the Standard deviation of the portfolio’s excess return.

#### Information ratio (IR)

Like the Sharpe Ratio, the Information Ratio compares relative return with relative risk, and its expression is as follows:

7 $$Information Ratio = \frac{{R_{p} - R_{M} }}{Tracking Error}$$

where *Rp* is the return of the portfolio; *R*_*M*_ the return of benchmark and the tracking error the standard deviation of difference between portfolio and benchmark returns. This IR is studied by Gardijan and Krišto (2017) and Yarovaya et al. (2020).

#### Treynor ratio

Like the Sharpe Ratio, the Treynor Ratio, proposed by Treynor in 1965, measures excess return relative to risk, only differing in the denominator in the two ratios, where the Treynor ratio uses the portfolio's beta (systematic risk) (Maruti and Shivaji 2013). We can thus express:

8 $$Treynor Ratio = \frac{{R_{p} - R_{f} }}{{\beta_{p} }}$$

where *Rp* is the return of the portfolio; *Rf* the risk-free rate and $\beta_{p}$ is the portfolio’ beta.

#### Sortino ratio

Sortino and Price (1994) emphasize that the Treynor and Sharpe ratios have limitations, as they assume that all deviations from the investors' objective rate constitute a risk for the investor. The Sortino ratio will only penalize returns below the intended target of the required rate of return; on the other hand, The Sharpe ratio penalizes both upside and downside volatility equally (Walavalkar et al. 2020). The expression of the Sortino Ratio is:

9 $$Sortino Ratio = \frac{{R_{p} - R_{f} }}{{\sigma_{d} }}$$

where *Rp* is the return of the portfolio; *Rf* the risk-free rate and $\sigma_{d}$ is the Standard deviation of the downside.

### Specific funds’ Measures

#### Net Asset Value

The Net Asset Value is the ratio between the net value of all assets and the number of outstanding units of the fund (Walavalkar et al. 2020). This value is calculated daily and whenever an investor wants their money back, the fund buys the units at the unit price (Macey 2011). Thus, this value is the price at which investors can buy units or sell them back to the fund (Pellegrini et al. 2017). The expression is:

10 $$Net Asset Value = \frac{Net assets of the scheme}{{Number of units outstanding}}$$

where, Net assets of the scheme are equal to: Market value of investment + Receivables + Other accrued income + Other assets − Accrued expenses − Other payables − other liabilities (Maruti and Shivaji 2013).

#### Expense ratio

The Centre for Research and Security Prices (2022) defines expense ratio as the fund’s operating expenses paid by shareholders to the total investment. These expenses are custodial fees, management fees, marketing expenses, and others (Gouveia et al. 2018). The expense ratio is expressed in the percentage of the investment (Walavalkar et al. 2020). In literature, expense ratio is used as input in efficiency evaluation as the managers and investors want to reduce this ratio (see the summary table of the literature by Henriques et al. 2022).

## Methodological framework

### Data selection and variables/factors

This study aims to analyze the efficiency of investment funds in three sectors affected by the COVID-19 pandemic—technology, healthcare, and the consumer cyclical. For the choice of funds, we analyzed the rankings provided by a digital media company dedicated to helping citizens, consumers, investors—The U.S. News & World Report. Several studies in the literature analyze their rankings too (e.g., Alsmadi et al. 2020; Prasad and Goldstein 2014; Rank 2008). Thus, this study examined 44 technological funds, 30 healthcare funds, and 7 consumer cycle funds ranked by this agency, as expressed in Table A1—Appendix.^1^

Regarding the variables, we collected them in the Eikon and the Morningstar. We have available data from the last 5 years in these two databases, so we analyze 1 year (2020), 3 years (2018–2020), and 5 years (2016–2020) data. The funds remained active during the period under review, which indicates no bias in the results.

It should be noted that the model is not applied separately to each sector, it was always applied to the same 81 funds, considering 1 year, 3 years, and 5 years. We cannot run the Value-Based DEA for just 7 funds because of the diminished discriminatory power. However, to evaluate these 7 funds, we could introduce restrictions on the weights of inputs and outputs, as in Gouveia et al. (2016), and improve the discrimination of DMUs. This is one of the ways to overcome this pitfall according to the authors Dyson et al. (2001).

To select the variables to use as factors (inputs and outputs) in our methodology DEA, we employed a Principal Components Analysis, explained below.

#### Principal component analysis

The principal component analysis is a multivariate statistical technique that uses an orthogonal transformation to transform a set of original, possibly correlated, variables into linearly uncorrelated variables called principal components (Hair et al. 2009).

Initially, we collected Net Asset Values (NAV), Expense Ratio (ExpRatio), Alpha, Annualized Standard Deviation (StDevia), Beta, Correlation (Correl), Information Ratio (IR), Max Drawdown (MaxDrawd), Risk/Reward Ratio (RRRatio), R-Square, Sharpe Ratio, Sortino, Tracking Error (TrackError), Treynor Ratio, Value at Risk Normal (VAR), Value at Risk Normal ETL (VARETL), Value at Risk Quantile (VARQuantile) and Variance as factors used by several authors as inputs and outputs in DEA methodology.

Given the above, and taking into account the measures that the literature most uses to analyze the efficiency of funds, we choose to consider Variance, Standard deviation, Beta, and R-square as inputs. We compute PCA to aggregate these variables into principal components. As we can see in Table 2, we obtain three different principal components. The first Component privileges risk measures; component two highlights the return with relief for the Sharpe and R/R ratio and the Alpha, and component three with the high explanatory power of the correlations and R-square variables. Our choice is in line with Tuzcu and Ertugay (2020) because the risk measures should reflect the overall risk as well as the positive impacts of portfolio diversification.

**Table 2:** PCA

Variable	Component 1	Component 2	Component 3	Unexplained
NAV				0.9294
ExpRatio				0.6513
Alpha		0.3540		0.1312
StDevia	− 0.3402			0.02465
Beta	− 0.3042			0.2218
Correl			0.5880	0.02335
IR		0.3461		0.3556
MaxDrawd				0.3834
RRRatio		0.3660		0.1045
RSquare			0.5791	0.02506
Sharpe		0.3718		0.08883
Sortino				0.4896
TrackError	− 0.3090			0.04848
Treynor		0.3516		0.3409
VAR	0.3976			0.0328
VARETL	0.3943			0.02495
VARQuantile				0.3979
Variance	− 0.3420			0.05209

As outputs, component two stands out, considering the Sharpe ratio and Alpha. In the model specification some of the inputs are part of the outputs. But, there is no problem in the specification of the model even if we are working with inputs that are already part of the outputs. By using PCA the possible collinearity problem is solved by creating an index composed by the variables used (Table 2), which will be uncorrelated with the model inputs and outputs (Table 3) allowing us to use the model specification.

**Table 3:** Inputs and Outputs

Factors to minimize (inputs)	Factors to maximize (outputs)
Variance	Sharpe Ratio
Annualized Standard deviation	
Beta	Alpha
R-squared	

Table 3 summarizes the model and the chosen inputs and outputs, which will be used to measure the efficiency of the three types of investment funds.

### Methodology

DEA models were first introduced by Charnes et al. (1978) under the consideration of constant returns to scale, latter the variable returns to scale (VRS) assumption was enabled in the model proposed by (Banker et al. 1984). Either way, according to these models, the relative efficiency of a DMU is evaluated by the ratio between the weighted sum of its outputs and the weighted sum of its inputs. The Value Based DEA model is VRS, suitable for accommodating different fund sizes. However, when making the transformation to the value scale, the value zero was assigned to the worst original performance and the value 1 to the best original performance in each factor. This was done for each period under evaluation. Thus, the issue of outliers is solved.

There is another frequently used model, the DEA additive model, suggested by Charnes et al. (1985), nevertheless, this modeling approach does not provide a score of (in)efficiency for those DMUs that fail to pass the test of Pareto–Koopmans efficiency. What we mean is this, the DEA additive model does not return a measure of (in)efficiency with an intuitive interpretation. Later Ali et al. (1995) proposed a variant of the DEA additive model with a weighted sum in the objective function, but the components of the vector of weights are considered as given constants. However, it is difficult to establish this vector a priori, and the projections of inefficient DMUs in any of the additive models are dependent on the scales used on each criterion.

Gouveia et al. (2008) developed the Value-Based DEA model which overcomes the problem of scales and the lack of interpretation of the value returned by the weighted additive model (Ali et al. 1995). The Value-Based DEA combines DEA and MCDA as a way of incorporating the preference information provided by DMUs into the analysis, converting the inputs and outputs into a value scale.

Note that, in the Value-Based DEA method, DMUs play the role of alternatives to be compared using concepts developed in the area of MCDA under imprecise information (Gouveia et al. 2008). This approach considers a set of acceptable vectors for the scale coefficients, rather than a single vector. Thus, the overall value of each alternative is no longer precisely determined. In this context of additive aggregation with imprecise weights, the idea of using the well-known min–max regret rule (Bell 1982) have been suggested as a way to compare the alternatives (Salo and Hämäläinen 2001). In the Value-Based DEA method proposed for this study, the scale coefficients (weights) that, for each alternative, minimize the value difference to the best alternative, are found according to the min–max regret rule, which gives an intuitive meaning to the efficiency measure assigned to each DMU (loss of value). Assuming the presence of external uncertainty in the DMU coefficients in each factor (input or output), the proposed method includes the concept of super-efficiency in order to provide the robustness analysis of any DMU (Gouveia et al. 2013). The Value-Based DEA is a photograph of year-end (annual) values where there are no inflows and outflows of assets. It is a static model that treats funds according to their composition at the end of each year.

It should be noted that isotonicity relations between inputs and outputs assumed in DEA, i.e., an increase in an input should not lead to a decrease in an output (Golany and Roll 1989), result in a positive correlation between inputs and outputs. However, this is associated with production relations that are governing the DMUs to be analysed and the criteria that have been either explicitly proposed or implicitly used for the selection of inputs and outputs. In our study, we consider the preference of decision-makers (hypothetical investors) towards input or output factors as attributes to minimize or maximize, respectively. After the performance measures in DEA factors are converted into value functions to be maximized, the isotonicity problem vanishes. That transformation considers that it is better to have less risk and more return, even if the lower risk does not mean having more return.

Considering that we have a set of *n* alternatives (DMUs) to be evaluated $\left\{{DMU}_{j}:j=1,\dots ,n\right\}$ according to a set of *q* criteria, with *q* = *m* + *p*, ${x}_{ij} \left(i=1,\dots ,m\right)$ to be minimized, and ${y}_{rj} \left(r=1,\dots ,p\right)$ to be maximized. The conversion consists of, using Multi-Attribute Utility Theory (MAUT) concepts, to build partial value functions $\left\{{{v}_{c}(DMU}_{j}), c=1,\dots ,q, j=1,\dots ,n\right\}$. Each of them is defined in the range [0,1] considering that for each factor *c* the worst performance ${p}_{cj}$, $j=1,\dots ,n,$ has the value 0 and the best performance ${p}_{cj}$, $j=1,\dots ,n,$ has the value 1, resulting in a maximization of all criteria. After that, they are gathered into a global value function, $V\left({DMU}_{j}\right)={\sum }_{c=1}^{q}{w}_{c}{v}_{c}\left({DMU}_{j}\right)$, where ${w}_{c}\ge 0$, ∀*c* = 1,…,*q* and ${\sum }_{c=1}^{q}{w}_{c}=1$ (by convention). The weights ${w}_{1},\dots ,{w}_{q}$ considered in the additive value function are the scale coefficients and are settled in a way that each alternative minimizes the value difference to the best alternative, according to the min–max regret rule (Bell 1982).

The Value-Based DEA method comprises two phases after all factors have been converted into a value scale.

*Phase 1* Compute the efficiency measure, ${d}_{k}^{*}$, for each ${DMU}_{k}$ (k = 1,…,n), and the corresponding weighting vector ${w}_{k}^{*}$ by solving the linear problem (11).

*Phase 2* If ${d}_{k}^{*}\ge 0$ then solve the “weighted additive” problem (12), using the optimal weighting vector resulting from Phase 1, ${w}_{k}^{*}$, and determine the corresponding projected point of the ${DMU}_{k}$ under evaluation.

Gouveia et al. (2013) included the concept of super-efficiency (Andersen and Petersen 1993) in formulation (12) to accommodate the discrimination of efficient DMUs.

$$\underset{{d}_{k},w}{\mathrm{min}}{d}_{k}$$

$$s.t. \sum_{c=1}^{q}{w}_{c}{v}_{c}\left({DMU}_{j}\right)-\sum_{c=1}^{q}{w}_{c}{v}_{c}\left({DMU}_{k}\right)\le {d}_{k}, j=1,\dots ,n;j\ne k$$

$$\sum_{c=1}^{q}{w}_{c}=1$$

11 $${ }w_{c} \ge 0, \forall c = 1, \ldots ,q$$

The optimal value of the objective function, ${d}_{k}^{*}$, for each DMU *k* (*k* = *1,…,n*) and the corresponding weighting vector are the variables in the linear problem (13). The score ${d}_{k}^{*}$ is the distance defined by the value difference to the best of all DMUs (note that the best DMU will also depend on *w*), excluding itself from the reference set. If ${d}_{k}^{*}$ is negative, then the DMU *k* under evaluation is efficient, and it is possible to rank these efficient units taking into account that the more negative the value${d}_{k}^{*}$, the more efficient the DMU.

If a DMU has a non-negative score, ${d}_{k}^{*},$ then the DMU is inefficient and a target can be computed solving the linear problem (12):

$$\underset{\lambda ,s}{\mathrm{min}}{z}_{k}=-\sum_{c=1}^{q}{w}_{c}^{*}{s}_{c}$$

$$s.t.\sum_{j=1,j\ne k}^{n}{\lambda }_{j}{v}_{c}\left({DMU}_{j}\right)-{s}_{c} = {v}_{c}\left({DMU}_{k}\right), c=1,\dots ,q$$

$$\sum_{j=1,j\ne k}^{n}{\lambda }_{j}=1$$

12 $$\lambda_{j} , s_{c} \ge 0, j = 1, \ldots ,k - 1,k + 1, \ldots ,n; c = 1, \ldots ,q$$

A convex combination of the value score vectors associated with the *n*-1 DMUs is expressed by the variables λ*j*, *j* = 1,…,*k*-1,*k* + 1,…,*n*. The set of efficient DMUs (it could be a single one) that define convex combination with λ*j* > 0 are called the “peers” of DMU *k* under evaluation. This convex combination corresponds to a point on the efficient frontier that is better than DMU *k* by a difference of the value of ${s}_{c}$ (slack) in each criterion *c*.

## Results

### Descriptive statistics

Table 4 presents the main descriptive statistics (mean, standard deviation, minimum, and maximum) of the factors used in this study between 2016 and 2020.

**Table 4:** Summary Statistics

Factors	Mean	St. Deviation	Min	Max
Variance	30.829	17.210	14.760	152.192
Standard Deviation	18.781	4.176	13.309	42.735
Beta	1.027	0.138	0.570	1.627
R-Square	0.830	0.129	0.278	0.995
Sharpe Ratio	0.292	0.097	0.027	0.461
Alpha	0.130	0.375	− 1.069	1.401

As can be seen, the funds have an Annualized standard deviation different from zero, representing portfolios with some risk. The Beta on average is close to one, which indicates that the net worth of the funds in the analysis will evolve as their benchmark. The R-square is in line with Beta as it correlates closer to 1 with its benchmark. Regarding the outputs, the Alpha is positive, which indicates the manager is making a return from the funds under study. The Sharpe ratio, being greater than zero, shows that investors attach importance to risk-adjusted returns; this ratio does not present negative values, so there are no inconsistent assessments (Agudo and Marzal 2004).

### Value functions

Considering that the value ${p}_{cj}$ is the performance of DMU *j* in factor *c*, the inputs and outputs performances are linearly converted into “values”.

The values for each DMU (fund) were computed using:

13 $$v_{c} \left( {DMU_{j} } \right) = \left\{ {\begin{array}{*{20}c} {\frac{{p_{cj} - M_{c}^{L} }}{{M_{c}^{U} - M_{c}^{L} }}, if factor c is an output} \\ {\frac{{M_{c}^{U} - p_{cj} }}{{M_{c}^{U} - M_{c}^{L} }}, if factor c is an input} \\ \end{array} } \right., j = 1, \ldots ,n;c = 1, \ldots ,q$$

where the values ${M}_{c}^{L}<min\left\{{p}_{cj},j=1,\dots ,n\right\}$ and ${M}_{c}^{U}>max\left\{{p}_{cj},j=1,\dots ,n\right\}$, for each *c* = *1,…,q,* were considered according to the minimum and the maximum depicted in Table 4.

With this type of transformation, the Valued-based DEA overcomes one of the most known limitations of the classic DEA models, the data present negative or null values, by converting the performances on each factor into a value scale. The *q* value functions are defined such that the worst level has value 0 and the best level has value 1. Thus, after being converted into values, all factors are treated as outputs to be maximized.

### Results and discussion

#### Efficiency

We will present below the results for 1 year (2020—COVID-19 year), three years (2018–2020), and 5 years (2016–2020). The number of efficient funds for each of these periods was analyzed, as the main factors that explain their efficiency. As we express in Tables 5, 6, and 7, the efficient funds have a negative d*. In Tables A2, A3, and A4 (in Appendix), we present the d* values for all funds studied and each period. These tables are ordered by sector and sorted in descending order of efficiency.

**Table 5:** Results of value-based DEA for the 9 efficient funds for 1 year (2020)

Fund	Sector	${d}^{*}$	${w}_{VAR}^{*}$	${w}_{SD}^{*}$	${w}_{BETA}^{*}$	${w}_{RSQ}^{*}$	${w}_{SHARPE}^{*}$	${w}_{ALPHA}^{*}$
78	Consumer Cyclical	− 0.127	0.000	0.000	0.000	**0.592**	**0.408**	0.000
6	Healthcare	− 0.048	0.000	**0.932**	0.000	0.000	0.068	0.000
21	Healthcare	− 0.038	0.000	**0.702**	0.000	0.152	0.000	0.146
31	Healthcare	− 0.025	0.000	0.000	0.000	**0.599**	0.000	**0.401**
13	Tecnhonology	− 0.118	0.000	0.000	0.000	0.068	0.000	**0.932**
25	Tecnhonology	− 0.020	0.000	0.299	0.000	0.000	0.000	**0.701**
34	Tecnhonology	− 0.002	0.000	0.000	**0.767**	0.002	0.000	**0.231**
40	Tecnhonology	− 0.043	0.000	0.000	**0.977**	0.000	0.023	0.000
56	Tecnhonology	− 0.002	**0.629**	**0.185**	0.000	0.000	**0.185**	0.000

**Table 6:** Results of value-based DEA for the 19 efficient funds for 3 years

Fund	Sector	${d}^{*}$	${w}_{VAR}^{*}$	${w}_{SD}^{*}$	${w}_{BETA}^{*}$	${w}_{RSQ}^{*}$	${w}_{SHARPE}^{*}$	${w}_{ALPHA}^{*}$
78	Consumer Cyclical	− 0.020	0.298	0.000	**0.421**	0.000	0.000	0.282
4	Healthcare	− 0.035	0.000	**0.576**	0.101	**0.323**	0.000	0.000
20	Healthcare	− 0.013	0.000	**0.818**	0.000	0.000	0.182	0.000
21	Healthcare	− 0.059	0.000	**0.391**	0.000	**0.251**	0.188	0.169
27	Healthcare	− 0.154	0.000	0.000	0.194	**0.806**	0.000	0.000
31	Healthcare	− 0.007	**0.469**	0.000	0.037	**0.494**	0.000	0.000
39	Healthcare	− 0.002	**0.753**	0.000	0.000	0.000	0.247	0.000
41	Healthcare	− 0.022	0.000	**0.472**	0.000	**0.385**	0.012	0.131
46	Healthcare	− 0.016	0.000	**0.328**	**0.379**	0.134	0.159	0.000
65	Healthcare	− 0.023	0.185	0.000	**0.524**	0.064	0.000	0.228
7	Tecnhonology	− 0.006	0.000	0.334	**0.408**	0.000	0.258	0.000
13	Tecnhonology	− 0.393	0.000	0.000	0.000	0.000	0.000	**1.000**
22	Tecnhonology	0.000	0.273	0.000	**0.526**	0.000	0.139	0.062
23	Tecnhonology	− 0.044	0.000	0.000	0.000	0.097	**0.822**	0.081
25	Tecnhonology	− 0.015	0.093	0.000	**0.442**	0.163	0.302	0.000
26	Tecnhonology	− 0.003	0.268	0.000	**0.514**	0.033	0.185	0.000
33	Tecnhonology	− 0.005	0.000	0.245	**0.568**	0.019	0.000	0.168
40	Tecnhonology	− 0.116	0.000	0.000	**0.836**	0.000	0.164	0.000
3	Tecnhonology/Healthcare	− 0.011	0.000	0.000	0.000	0.585	0.000	0.415

**Table 7:** Results of value-based DEA for the 19 efficient funds for 5 years

Fund	Sector	${d}^{*}$	${w}_{VAR}^{*}$	${w}_{SD}^{*}$	${w}_{BETA}^{*}$	${w}_{RSQ}^{*}$	${w}_{SHARPE}^{*}$	${w}_{ALPHA}^{*}$
4	Healthcare	− 0.013	0.000	**0.707**	0.000	0.293	0.000	0.000
12	Healthcare	− 0.001	0.000	**0.765**	0.000	0.142	0.093	0.000
21	Healthcare	− 0.021	0.000	**0.394**	0.020	0.373	0.188	0.025
27	Healthcare	− 0.085	0.000	0.000	0.123	**0.641**	0.000	0.236
39	Healthcare	− 0.004	0.000	**0.841**	0.000	0.000	0.159	0.000
46	Healthcare	− 0.001	0.000	**0.756**	0.000	0.153	0.091	0.000
62	Healthcare	− 0.007	0.000	**1.000**	0.000	0.000	0.000	0.000
65	Healthcare	− 0.002	0.420	0.000	0.000	**0.580**	0.000	0.000
7	Tecnhonology	− 0.006	0.000	0.346	**0.462**	0.000	0.193	0.000
13	Tecnhonology	− 0.188	0.000	0.000	0.000	0.000	0.000	**1.000**
23	Tecnhonology	− 0.019	0.000	0.000	0.000	0.024	**0.534**	0.443
25	Tecnhonology	− 0.017	0.000	0.112	**0.366**	0.243	0.280	0.000
33	Tecnhonology	− 0.009	0.000	0.184	**0.316**	0.185	0.163	0.152
40	Tecnhonology	− 0.103	0.000	0.000	**1.000**	0.000	0.000	0.000
54	Tecnhonology	− 0.002	**0.624**	0.000	0.287	0.000	0.088	0.000
55	Tecnhonology	− 0.034	0.000	0.000	0.059	**0.464**	**0.476**	0.000
63	Tecnhonology	− 0.001	0.000	0.000	0.000	0.000	**0.860**	0.140
70	Tecnhonology	− 0.009	0.000	0.291	0.055	**0.307**	**0.304**	0.042
71	Tecnhonology	− 0.031	0.000	**0.476**	0.000	0.000	0.029	**0.495**
3	Tecnhonology/Healthcare	− 0.225	0.000	0.000	0.000	1.000	0.000	0.000

##### One year

As expressed in Table 5, in 2020, the year of COVID-19, there are only 9 efficient funds in a total of 81 funds. From the health sector, it is concluded that two of the three efficient funds assign a greater weight to the Standard deviation. This result is in line with that obtained by Chen et al. (2018) who find that investors use healthcare funds to hedge potential market risks. The manager is aware that the composition of his portfolio is of low risk, probably to meet the needs of the profile of investors.

The Alpha factor, a risk-adjusted performance measure, is the biggest contributor to the efficiency of two of the five efficient funds in the technology sector. Fund 13 assigns about 94% of the weight to this indicator. In two other funds, the greatest weight is assigned to Beta, a measure of risk, which measures the sensitivity of that portfolio with its benchmark. Fund 40 assigns about 98% of the weight to the Beta, suggesting that the investor with this fund knows how to be less risky than the market. Finally, fund 56 gives importance to both risk and performance measures. Risk measures are intrinsic measures to the composition of the fund and not to market risk, so the manager is aware of the specificity of this type of fund which, despite the risk, also weighs performance. Generally, the higher the Sharpe ratio of a portfolio, the better its risk-adjusted-performance. But the Sharpe ratio can also help explain whether a portfolio's excess returns are due to smart management investment decisions or the result of too much risk. In this case, although the weights of the Standard deviation and the Sharpe index are the same, there is a high weighting in the fund's Variance, which may suggest that it is a specific technological fund for potential players investors.

Concerning the consumer sector, there is only one efficient fund in 2020, which is not surprising given the unemployment figures and society's lack of resources to have purchasing power. The factors that support its efficiency are the R -Square and the Sharpe Index. In this case, fund managers try to explain their movements by their benchmark movements and give importance to risk-adjusted returns.

Furthermore, the lack of efficiency in this sector can be explained by the fact that investors have less disposable income to invest in a sector of this nature, cyclical and with serious consequences due to the COVID-19 crisis (Gejalakshmi and Azhagaiah 2017).

##### Three years

Considering the data for 3 years, the period immediately before the COVID-19 crisis, the number of efficient funds was much higher, 19 against 9 (see Table 6). It seems that nothing predicted the crisis that would happen with the pandemic. In the health sector, and as already shown for the year 2020, it appears that seven of the nine efficient funds attribute greater weight to the Standard deviation or to the Variance, which are measures that represent the total risk of the portfolio. Three funds are efficient because the manager recognizes that they are less risky than the market itself and that is why they assign weights to the Beta. Managers recognize that these funds are low-risk.

As we can see, in this sector, none of the funds attaches weight to performance indicators. R-squared is not a measure of the performance of a portfolio. Rather, it measures the correlation of the portfolio’s returns to the benchmark's returns. Thus, by recognizing high weights to the R^2^, managers are showing that the funds they manage do not deviate from the benchmark. They try to explain their movements by the movements of their benchmark. Moreover, investors choose this sector as a hedge against the risk of a market downturn (Chen et al. 2018).

In the technology sector, the managers of these funds give more consideration to the Beta indicator, which represents the systematic risk that cannot be diversified, that is, these managers know that the composition of their portfolios is better than that of other sectors and that they should add more assets to the portfolio no longer reduce the Beta. Once again, fund 40 is the one that most weights the Beta with around 83%. Seven of the funds’ weigh the Standard deviation and Variance showing once again, as in the healthcare sector, that the manager considers that the specific risk of these funds is not high. Fund 20 even considers that risk is the most important factor for its efficiency, which means that the manager is confident that it is a low-risk fund.

It should be noted that the efficiency of fund 13, the most efficient technology fund, comes only from Alpha. Alpha is used as an effective measure of performance, indicating when a manager's strategy has managed to outperform the market's return over a while. This means that in this period 2018–2020 this fund beats the market in terms of returns.

Managers of fund 23, the third most efficient technological fund, assign greater importance to the Sharpe ratio, a measure of return used to compare the performance of investment managers by adjusting risk. In this sector and for this period, it seems that no fund is in the interest of a rational investor, who takes into account the risk-return binomial. Efficient funds either weigh risk or return individually.

As verified for the year 2020, there is also an efficient fund in the area of consumption and an efficient fund that is from both the technology and health sectors. Thus, contrary to what happened in 2020, managers in fund 78 give importance to market risk and in fund 3 they distribute the weight between R-Square and Alpha in the health and technology sector.

##### Five years

Considering the data for 5 years, there are 20 efficient funds (Table 7), one more efficient fund compared with the data for 3 years.

During this period, efficient funds in the health sector attributed almost exclusively weight to the Standard deviation. It should be noted that fund 62 is efficient as it assigns 100% weight to this indicator. This result suggests that the manager knows that the fund's specific risk is very low and it is in line with those obtained in the other periods considered. There are still two funds that weigh the R^2^ to be efficient, without going much beyond 60%.

In the technological sector, and compared to other periods, there is greater dispersion in the weights attributed to each factor. It is highlighted that five funds assign more than 70% weight to the Standard deviation, and fund 62 even assigns 100%. Once again the fund 40 is efficient at giving 100% weight to the Beta. Fund 13 also maintains the same result as in the previous period when assigning 100% weight to Alpha. The other performance measure, the Sharpe ratio, was chosen by four funds to be efficient but only fund 63 gives a very high weight, around 86%. As seen in the 2018 to 2020 period, fund 3, which belongs to the technological and healthcare sectors, remains efficient, attributing all its efficiency to indicator R-Square.

#### Slacks

##### One year

By solving the linear problem from Phase 2 [formulation (12)] for inefficient DMUs (funds), the value of the slack variables is obtained. The solution is an efficiency target (projection) proposal for each inefficient DMU (fund). To reach the efficiency state and equalize the peers that serve as a reference, these inefficient DMUs have to change their value in each factor by the value indicated by s*.

Table 8 shows for the inefficient funds the values of slacks and the peers that each one chose on the frontier of efficiency for the year 2020. It must be noted that fund 6 is chosen 48 times by inefficient funds as a reference fund at the efficient frontier. Funds 21 and 56 are chosen by 28 and 23 inefficient funds, respectively, as benchmarks. The main characteristics of the efficient funds chosen are that they most often highlight the greater importance of Standard Deviation, R-Square, and Alpha factors.

**Table 8:** Results of Phase 2 of the value-based DEA for the inefficient funds for 1 year

Fund	Sector	${s}_{VAR}^{*}$	${s}_{SD}^{*}$	${s}_{BETA}^{*}$	${s}_{RSQ}^{*}$	${s}_{SHARPE}^{*}$	${s}_{ALPHA}^{*}$	Peers
18	Consumer Cyclical	0.113	0.221	0.180	0.034	0.000	0.102	6, 56
67	Consumer Cyclical	0.150	0.259	0.188	0.000	0.153	0.212	6, 21
77	Consumer Cyclical	0.021	0.049	0.033	0.000	0.000	0.021	6, 21,56
79	Consumer Cyclical	0.030	0.057	0.056	0.000	0.120	0.082	21, 56
80	Consumer Cyclical	0.127	0.185	0.218	0.000	0.154	0.163	21, 25
81	Consumer Cyclical	0.011	0.022	0.022	0.000	0.068	0.062	21, 56
1	Healthcare	0.035	0.086	0.100	0.000	0.139	0.044	6, 21
2	Healthcare	0.015	0.038	0.046	0.000	0.494	0.324	6, 21
4	Healthcare	0.050	0.115	0.138	0.000	0.561	0.399	6, 21
9	Healthcare	0.044	0.088	0.082	0.000	0.238	0.096	21,31
12	Healthcare	0.015	0.041	0.075	0.154	0.244	0.152	6
16	Healthcare	0.011	0.031	0.065	0.159	0.277	0.172	6
20	Healthcare	0.014	0.040	0.079	0.174	0.304	0.193	6
24	Healthcare	0.014	0.012	0.012	0.147	0.216	0.339	13
27	Healthcare	0.102	0.142	0.037	0.085	0.000	0.256	31, 78
28	Healthcare	0.003	0.007	0.026	0.079	0.305	0.222	21
39	Healthcare	0.019	0.051	0.091	0.161	0.212	0.131	6
41	Healthcare	0.009	0.021	0.028	0.000	0.212	0.122	21, 31
43	Healthcare	0.019	0.052	0.090	0.167	0.130	0.077	6
44	Healthcare	0.028	0.072	0.083	0.000	0.270	0.143	6
46	Healthcare	0.006	0.019	0.031	0.000	0.448	0.266	6, 21
47	Healthcare	0.015	0.043	0.064	0.085	0.120	0.060	6
48	Healthcare	0.022	0.058	0.104	0.182	0.378	0.250	6
49	Healthcare	0.018	0.050	0.102	0.255	0.158	0.110	6
50	Healthcare	0.014	0.038	0.046	0.006	0.034	0.000	6, 21
51	Healthcare	0.018	0.050	0.089	0.170	0.233	0.147	6
62	Healthcare	0.015	0.041	0.084	0.190	0.263	0.169	6
64	Healthcare	0.000	0.000	0.009	0.008	0.047	0.046	21, 31
65	Healthcare	0.078	0.152	0.059	0.000	0.176	0.402	6, 31
68	Healthcare	0.031	0.080	0.116	0.102	0.335	0.210	6
69	Healthcare	0.000	0.004	0.056	0.109	0.169	0.157	21, 31
72	Healthcare	0.016	0.045	0.067	0.084	0.114	0.055	6
74	Healthcare	0.028	0.073	0.112	0.122	0.384	0.248	6
3	Tecnhonology	0.830	0.713	0.836	0.326	0.000	0.361	31, 78
5	Tecnhonology	0.092	0.177	0.103	0.000	0.000	0.004	6, 21, 56
7	Tecnhonology	0.047	0.105	0.070	0.155	0.000	0.091	6, 56
8	Tecnhonology	0.038	0.094	0.088	0.184	0.000	0.162	6, 56
10	Tecnhonology	0.095	0.153	0.125	0.000	0.302	0.249	21, 56
11	Tecnhonology	0.060	0.092	0.082	0.256	0.000	0.064	21, 78
14	Tecnhonology	0.079	0.170	0.111	0.175	0.082	0.206	6
15	Tecnhonology	0.047	0.079	0.043	0.000	0.148	0.019	56, 78
17	Tecnhonology	0.076	0.165	0.101	0.188	0.022	0.152	6
19	Tecnhonology	0.100	0.193	0.093	0.000	0.000	0.053	6, 21, 31
22	Tecnhonology	0.026	0.047	0.051	0.021	0.357	0.304	56
23	Tecnhonology	0.123	0.231	0.129	0.000	0.000	0.022	6, 21, 56
26	Tecnhonology	0.101	0.204	0.105	0.211	0.133	0.169	6
29	Tecnhonology	0.058	0.098	0.057	0.064	0.258	0.108	56
30	Tecnhonology	0.128	0.244	0.153	0.061	0.041	0.121	6
32	Tecnhonology	0.176	0.307	0.280	0.068	0.283	0.470	6
33	Tecnhonology	0.102	0.196	0.157	0.000	0.310	0.598	6, 21
35	Tecnhonology	0.028	0.047	0.021	0.000	0.143	0.048	25, 78
36	Tecnhonology	0.069	0.149	0.101	0.184	0.000	0.114	6, 56
37	Tecnhonology	0.062	0.140	0.086	0.184	0.000	0.128	6, 56
38	Tecnhonology	0.000	0.000	0.000	0.006	0.006	0.009	21, 34
42	Tecnhonology	0.063	0.136	0.078	0.085	0.000	0.069	6, 56
45	Tecnhonology	0.057	0.110	0.080	0.546	0.176	0.278	21
52	Tecnhonology	0.230	0.364	0.257	0.000	0.170	0.254	6, 21
53	Tecnhonology	0.082	0.175	0.063	0.127	0.007	0.019	6
54	Tecnhonology	0.070	0.155	0.074	0.171	0.068	0.117	6
55	Tecnhonology	0.189	0.232	0.180	0.171	0.000	0.000	31, 40
57	Tecnhonology	0.045	0.076	0.042	0.000	0.141	0.017	56, 78
58	Tecnhonology	0.055	0.129	0.075	0.179	0.114	0.229	6
59	Tecnhonology	0.082	0.172	0.115	0.162	0.000	0.111	6, 56
60	Tecnhonology	0.101	0.194	0.155	0.000	0.324	0.609	6, 21
61	Tecnhonology	0.078	0.159	0.123	0.000	0.000	0.071	6, 21, 56
63	Tecnhonology	0.053	0.116	0.079	0.160	0.000	0.094	6, 56
66	Tecnhonology	0.031	0.054	0.029	0.000	0.159	0.061	56, 78
70	Tecnhonology	0.073	0.123	0.091	0.000	0.188	0.131	21, 56
71	Tecnhonology	0.047	0.114	0.093	0.184	0.058	0.097	6
73	Tecnhonology	0.035	0.064	0.055	0.000	0.211	0.158	21, 56
75	Tecnhonology	0.091	0.189	0.100	0.021	0.000	0.065	6, 56
76	Tecnhonology	0.146	0.268	0.157	0.235	0.080	0.116	6

All inefficient funds that elected fund 6, from the healthcare sector, as a benchmark only achieve it if they improve their performance by reducing all inputs and increasing the Alpha factor, as they present positive values associated with slacks in these factors. Besides, the funds that were chosen only once as part of the target on the efficiency frontier were funds 13, 34, and 40. All of them being funds from the technology sector, which made them efficient was the Alpha and Beta factors.

The factor that has the highest values associated with slacks, and therefore the factor that will necessarily have to perform best for most inefficient funds, is Alpha.

##### Three years

For 3 years, funds mainly choose fund 21 as a benchmark (23 times) followed by fund 20 (17 times), both from the healthcare sector. Improvements for inefficient funds that elect these as reference are more expressive in Standard Deviation and Alpha. This means that these inefficient funds should decrease Standard Deviation and increase the performance in Alpha because it is in these factors, the values of slacks are greater (see Table 9).

**Table 9:** Results of Phase 2 of the value-based DEA for the inefficient funds for 3 years

Fund	Sector	${s}_{VAR}^{*}$	${s}_{SD}^{*}$	${s}_{BETA}^{*}$	${s}_{RSQ}^{*}$	${s}_{SHARPE}^{*}$	${s}_{ALPHA}^{*}$	Peers
18	Consumer Cyclical	0.211	0.288	0.277	0.171	0.040	0.090	4
67	Consumer Cyclical	0.231	0.294	0.310	0.059	0.000	0.096	4, 33
77	Consumer Cyclical	0.000	0.023	0.142	0.180	0.015	0.000	40, 46
79	Consumer Cyclical	0.075	0.109	0.039	0.104	0.000	0.000	7, 46
80	Consumer Cyclical	0.208	0.237	0.184	0.199	0.023	0.006	41
81	Consumer Cyclical	0.138	0.199	0.071	0.162	0.049	0.010	46
1	Healthcare	0.038	0.056	0.071	0.000	0.000	0.006	4, 41
2	Healthcare	0.067	0.108	0.140	0.007	0.012	0.032	4
6	Healthcare	0.023	0.039	0.078	0.107	0.000	0.029	4, 21
9	Healthcare	0.096	0.133	0.166	0.000	0.019	0.032	4, 41
12	Healthcare	0.002	0.003	0.007	0.000	0.000	0.000	4, 20, 46
16	Healthcare	0.012	0.023	0.033	0.004	0.127	0.069	20
24	Healthcare	0.114	0.107	0.182	0.133	0.000	0.000	13, 40, 65
28	Healthcare	0.012	0.022	0.040	0.062	0.000	0.023	4, 21
43	Healthcare	0.050	0.083	0.135	0.090	0.000	0.020	4, 20
44	Healthcare	0.049	0.082	0.113	0.009	0.000	0.004	4, 20
47	Healthcare	0.052	0.085	0.119	0.000	0.072	0.040	20, 46
48	Healthcare	0.019	0.033	0.075	0.136	0.000	0.022	4, 20
49	Healthcare	0.028	0.050	0.080	0.050	0.043	0.029	20
50	Healthcare	0.044	0.069	0.110	0.082	0.000	0.021	4, 21
51	Healthcare	0.014	0.026	0.034	0.000	0.004	0.001	20, 21
62	Healthcare	0.005	0.008	0.017	0.016	0.146	0.080	20
64	Healthcare	0.005	0.013	0.093	0.000	0.124	0.163	27, 31
68	Healthcare	0.014	0.024	0.067	0.135	0.012	0.032	4
69	Healthcare	0.074	0.094	0.112	0.013	0.256	0.207	41
72	Healthcare	0.052	0.086	0.119	0.000	0.057	0.030	20, 46
74	Healthcare	0.014	0.025	0.072	0.153	0.120	0.097	4
5	Tecnhonology	0.052	0.080	0.040	0.107	0.000	0.126	21, 27
8	Tecnhonology	0.043	0.076	0.040	0.000	0.043	0.038	20, 78
10	Tecnhonology	0.113	0.148	0.045	0.159	0.169	0.216	21
11	Tecnhonology	0.000	0.024	0.134	0.214	0.000	0.276	13, 21
14	Tecnhonology	0.067	0.103	0.042	0.000	0.000	0.031	20, 26, 46
15	Tecnhonology	0.130	0.167	0.055	0.113	0.050	0.102	21
17	Tecnhonology	0.054	0.075	0.056	0.000	0.135	0.107	7, 46
19	Tecnhonology	0.077	0.100	0.103	0.184	0.000	0.173	21, 65
29	Tecnhonology	0.086	0.115	0.024	0.261	0.020	0.160	21
30	Tecnhonology	0.049	0.062	0.118	0.101	0.000	0.030	25, 65
32	Tecnhonology	0.076	0.098	0.047	0.184	0.000	0.000	21, 41, 65
34	Tecnhonology	0.164	0.202	0.111	0.000	0.113	0.161	21, 27,
35	Tecnhonology	0.144	0.182	0.074	0.000	0.040	0.087	21, 27
36	Tecnhonology	0.015	0.021	0.024	0.005	0.105	0.073	7
37	Tecnhonology	0.063	0.098	0.045	0.021	0.000	0.033	20, 26
38	Tecnhonology	0.000	0.024	0.135	0.139	0.000	0.277	13, 21, 27
42	Tecnhonology	0.039	0.052	0.046	0.000	0.147	0.105	7, 21
45	Tecnhonology	0.050	0.076	0.025	0.012	0.000	0.067	7, 20
52	Tecnhonology	0.127	0.163	0.059	0.000	0.025	0.000	13, 21, 27
53	Tecnhonology	0.000	0.022	0.179	0.128	0.000	0.109	26, 40, 46
54	Tecnhonology	0.002	0.005	0.000	0.000	0.000	0.072	7, 20, 26
55	Tecnhonology	0.000	0.024	0.048	0.012	0.000	0.204	13, 21, 27
56	Tecnhonology	0.041	0.054	0.072	0.000	0.092	0.027	25, 26
57	Tecnhonology	0.130	0.167	0.055	0.109	0.051	0.102	21
58	Tecnhonology	0.066	0.111	0.027	0.000	0.007	0.077	20, 40
59	Tecnhonology	0.042	0.055	0.062	0.006	0.189	0.134	7
60	Tecnhonology	0.000	0.000	0.001	0.003	0.033	0.026	33
61	Tecnhonology	0.007	0.008	0.206	0.188	0.000	0.232	65, 78
63	Tecnhonology	0.005	0.007	0.008	0.000	0.004	0.001	7
66	Tecnhonology	0.118	0.153	0.052	0.140	0.218	0.253	21
70	Tecnhonology	0.000	0.002	0.000	0.000	0.004	0.000	7, 13, 21, 25
71	Tecnhonology	0.047	0.077	0.038	0.048	0.046	0.000	20, 21
73	Tecnhonology	0.080	0.108	0.019	0.219	0.067	0.148	21
75	Tecnhonology	0.052	0.066	0.106	0.144	0.197	0.148	25
76	Tecnhonology	0.108	0.147	0.109	0.091	0.000	0.083	7, 46

Funds 3, 22, and 39 were not chosen as pairs by any of the funds classified as inefficient, and funds 23 and 31 were chosen only once.

##### Five years

In Table 10 there are the results obtained from phase 2 of the Value-Based DEA for 5 years. In this case, 21 funds choose fund 71 as the benchmark, followed by funds 7 and 62, which were chosen 15 times. The Standard Deviation and Alpha factors continue to be chosen as the characteristics associated with these funds that make them efficient and where there will have to be changed by the inefficient funds to be as good as the frontier funds they selected for the benchmark.

**Table 10:** Results of Phase 2 of the value-based DEA for the inefficient funds for 5 years

Fund	Sector	${s}_{VAR}^{*}$	${s}_{SD}^{*}$	${s}_{BETA}^{*}$	${s}_{RSQ}^{*}$	${s}_{SHARPE}^{*}$	${s}_{ALPHA}^{*}$	Peers
18	Consumer Cyclical	0.109	0.169	0.262	0.092	0.007	0.112	46
67	Consumer Cyclical	0.136	0.199	0.260	0.003	0.000	0.084	4, 21
77	Consumer Cyclical	0.077	0.128	0.044	0.005	0.000	0.000	7, 62, 71
78	Consumer Cyclical	0.032	0.052	0.121	0.163	0.000	0.000	13, 33, 71
79	Consumer Cyclical	0.068	0.109	0.013	0.035	0.000	0.001	62, 71
80	Consumer Cyclical	0.133	0.173	0.433	0.160	0.000	0.173	21, 33
81	Consumer Cyclical	0.079	0.130	0.048	0.000	0.000	0.000	7, 33, 62, 71
1	Healthcare	0.035	0.055	0.159	0.000	0.000	0.036	4, 21, 33
2	Healthcare	0.016	0.030	0.066	0.036	0.000	0.025	4, 21
6	Healthcare	0.024	0.042	0.104	0.052	0.000	0.012	46, 71
9	Healthcare	0.100	0.149	0.284	0.000	0.000	0.035	4, 21, 33
16	Healthcare	0.013	0.024	0.045	0.000	0.026	0.022	4, 62
20	Healthcare	0.005	0.010	0.020	0.000	0.000	0.001	4, 12, 62
24	Healthcare	0.000	0.000	0.068	0.102	0.137	0.296	13, 33
28	Healthcare	0.020	0.035	0.080	0.027	0.000	0.010	46, 71
31	Healthcare	0.090	0.121	0.394	0.000	0.034	0.144	33, 65
41	Healthcare	0.006	0.013	0.148	0.000	0.000	0.020	21, 33, 55
43	Healthcare	0.036	0.064	0.121	0.008	0.012	0.018	12
44	Healthcare	0.034	0.061	0.109	0.000	0.036	0.037	12, 46
47	Healthcare	0.026	0.044	0.122	0.085	0.000	0.021	46, 71
48	Healthcare	0.013	0.024	0.052	0.019	0.000	0.008	4, 62
49	Healthcare	0.012	0.023	0.056	0.041	0.000	0.007	39, 62
50	Healthcare	0.043	0.073	0.140	0.000	0.001	0.000	12, 46, 71
51	Healthcare	0.012	0.023	0.040	0.000	0.000	0.002	12, 39, 62
64	Healthcare	0.000	0.005	0.343	0.050	0.078	0.224	33, 65
68	Healthcare	0.010	0.020	0.042	0.000	0.057	0.047	4, 62
69	Healthcare	0.000	0.016	0.394	0.152	0.108	0.294	27, 33
72	Healthcare	0.034	0.060	0.115	0.000	0.000	0.004	12, 46, 71
74	Healthcare	0.009	0.016	0.076	0.113	0.044	0.061	4
5	Tecnhonology	0.000	0.003	0.052	0.034	0.132	0.187	55, 70
8	Tecnhonology	0.003	0.005	0.015	0.026	0.073	0.000	54, 71
10	Tecnhonology	0.088	0.132	0.121	0.054	0.000	0.150	46, 71
11	Tecnhonology	0.104	0.142	0.114	0.036	0.000	0.109	21, 70
14	Tecnhonology	0.007	0.011	0.069	0.069	0.000	0.084	7, 33
15	Tecnhonology	0.038	0.052	0.077	0.000	0.161	0.146	21, 70
17	Tecnhonology	0.057	0.093	0.074	0.000	0.000	0.110	7, 62, 71
19	Tecnhonology	0.086	0.131	0.110	0.030	0.000	0.150	46, 71
22	Tecnhonology	0.000	0.000	0.001	0.002	0.000	0.000	7, 33,54, 62
26	Tecnhonology	0.002	0.003	0.000	0.039	0.022	0.114	7, 33
29	Tecnhonology	0.043	0.063	0.080	0.035	0.092	0.134	7
30	Tecnhonology	0.000	0.003	0.048	0.048	0.094	0.135	55, 70
32	Tecnhonology	0.061	0.094	0.099	0.000	0.000	0.025	21, 46, 71
34	Tecnhonology	0.113	0.152	0.125	0.000	0.000	0.102	21, 55, 70
35	Tecnhonology	0.113	0.154	0.102	0.002	0.000	0.111	21, 70
36	Tecnhonology	0.011	0.017	0.032	0.009	0.075	0.061	7
37	Tecnhonology	0.036	0.060	0.040	0.000	0.000	0.079	7, 62, 71
38	Tecnhonology	0.120	0.161	0.124	0.000	0.001	0.111	21, 55
42	Tecnhonology	0.043	0.068	0.042	0.089	0.000	0.152	39, 71
45	Tecnhonology	0.023	0.038	0.015	0.039	0.000	0.108	7, 62
52	Tecnhonology	0.000	0.005	0.063	0.033	0.000	0.022	13, 21, 55
53	Tecnhonology	0.070	0.113	0.055	0.023	0.000	0.222	62, 71
56	Tecnhonology	0.017	0.026	0.056	0.000	0.090	0.047	21, 70
57	Tecnhonology	0.039	0.054	0.074	0.000	0.146	0.136	21, 70
58	Tecnhonology	0.010	0.019	0.000	0.007	0.000	0.039	7, 62
59	Tecnhonology	0.026	0.040	0.075	0.031	0.169	0.147	7
60	Tecnhonology	0.001	0.001	0.001	0.000	0.026	0.023	4, 33
61	Tecnhonology	0.091	0.139	0.165	0.011	0.000	0.032	46, 71
66	Tecnhonology	0.081	0.124	0.107	0.036	0.000	0.159	33, 65
73	Tecnhonology	0.034	0.052	0.067	0.000	0.129	0.119	7, 71
75	Tecnhonology	0.067	0.104	0.077	0.059	0.000	0.155	46, 71
76	Tecnhonology	0.053	0.077	0.101	0.042	0.117	0.159	7

The improvements of funds that chose fund 71 as a peer should be mostly through the reduction of inputs.

### General discussion

The results point out a decrease in the number of efficient funds in 2020, which can be justified by the COVID-19 pandemic crisis. The factors that determine the efficiency of funds in the health sector and the technology sector are quite distinct, although they have not undergone major changes in the three periods considered. The efficient health funds do not consider the profitability indicators in the periods before COVID-19 pandemics and in the COVID period, 2020, two funds consider Sharpe and Alpha, but with low weights, in the order of 40%. In this sector, it is always the Standard deviation that bears the greatest weight in all periods. In particular, for 5 years, fund 62 gives a weight of 100% to the Standard deviation, and four other funds weight that indicator at more than 75%.

In the technology sector, Beta and Alpha are generically the indicators with the most weight in the efficiency of funds, noting that fund 40 attributes 100% weight to this indicator in the periods before 2020 and around 98% in 2020. in the same way, the Alpha is weighted at 100% in the periods before the year 2020 and around 93% in 2020. Thus, technological funds beat the market in terms of returns and are less risky than the benchmark.

It is noted that for 5 years there is greater dispersion in the efficiency factors, probably because the manager has to protect himself from some market vulnerability.

Finally, health sector funds are the most used as a benchmark for inefficient funds, noting that to become efficient these funds have to assign more weight to the Standard deviation and Alpha. On the one hand, they should consider the specific risk of the fund, but also a performance measure that can identify whether the manager's investment strategy can outperform the market’s return.

From the point of view of the public interest in our work and for practitioners, our results specify that health and technology funds are an effective investment opportunity, considering different time horizons.

Health sector funds are perhaps better targeted at risk-averse investors, as they are safer, particularly in shorter periods and in crisis periods.

Funds in the technology sector are potential of more interest to investors who are not so risk-averse, rational investors who weigh risk but want higher levels of return. This result suggests that a risk-averse investor may reduce its exposure with funds from the healthcare sector while a rational investor would choose funds that beat the market.

Funds in the consumer goods sector will have less interest from investors or, as they are not very representative in our sample, they may have weak discriminatory power.

The results, therefore, show that the efficiency of funds depends on the economic cycle and that managers and investors adjust their risk level depending on whether the economy is booming or in recession, affecting their efficiency (Popescu and Xu 2017).

## Conclusion

The objective of this paper was to study the main factors that explain the efficiency of funds in three of the sectors most affected by COVID-19—the health, technology, and cyclical consumption sectors. To do this, and to respond to our research questions, first, a principal component analysis was carried out to determine the inputs and outputs to be used in the DEA methodology, which identifies the efficiency of the funds. Then, the efficiency of the funds for 1 year, 3 years, and 5 years was analyzed—in the year 2020, from 2018 to 2020, and between 2016 and 2020—seeking to identify the indicators that managers (based on investor preferences) consider most in an efficient fund.

In response to the first research question, there was a negative evolution in the number of efficient funds in the periods considered. The abrupt drop in 2020 should be highlighted, justified by the pandemic crisis, the COVID-19. Regarding our second and third research questions, the results point out that the funds' efficiency varies much more depending on the sector than with the periods considered. Our results also highlight that it is the health sector that is most often considered as a benchmark for inefficient funds both in the 1-year period and in the 3-year period. For the 5-year period, it is the technology sector that stands out as a benchmark for inefficient funds. This result suggests that while for a period further away from the pandemic, managers prioritize risk-adjusted performance measures, such as Alpha, for shorter periods, close to the pandemic and in a pandemic, the most important factor is the risk factor. We also found that the sectors with the most efficient funds are technology and healthcare.

The results of this study may be of interest to various stakeholders, namely investors and managers but also policymakers. Managers can understand which factors make funds more efficient, in addition to the traditional effects of diversification, and can make decisions that maximize investors' wealth. Investors can perceive that there are groups of funds that are more or less suited to their risk profile, and policymakers gain a better understanding of the behavior of this type of fund in different periods, namely in recessionary periods, which can help in the perception of the best possible regulatory decisions.

This work is not without limitations, since only three sectors of activity are being analyzed, it is necessary to be cautious in generalizing the obtained results. The value based DEA uses the L1 distance, which is considered pessimistic and, in addition, the efficiency score for a certain period of time is obtained without knowing the economic/social factors that may have influenced these results.

Furthermore, although we are using about 82% of the population data, there were restrictions on data collection which forced us to disregard some funds in our analysis. For example, in the consumer sector, only 7 funds were studied, which may have conditioned the discriminatory power of this sector, despite the population in the database containing only 9 funds.

Lastly, in this study some of the inputs are part of the outputs. Although PCA solve this problem, we recognize this limitation and in a future work this interacting role between inputs and outputs should be explored.

In future research, it would be also interesting to study more sectors of activity and realize the efficiency of funds after COVID-19 by using, for example, hybrid methodologies to treat inputs and outputs. As far as we know, the joint efficiency analysis of these sectors and the impact they suffered from the COVID-19 pandemic are new in the literature and our study will be important to managers, investors, and society, allowing them to react efficiently in a similar pandemic and/or crisis.

## See Tables 11, 12, 13 and 14

**Table 11:** Mutual funds under study

Name	Sector	Number of Fund
Rydex Leisure Fund	Consumer Cyclical	18
Fidelity Select Leisure Portfolio	Consumer Cyclical	67
Fidelity® Select Consumer Discret Port	Consumer Cyclical	77
Fidelity® Select Construction & Hsg Port	Consumer Cyclical	78
Fidelity® Select Retailing	Consumer Cyclical	79
Fidelity® Select Ports Automotive Port	Consumer Cyclical	80
Fidelity Advisor® Consumer Disctnry Fd	Consumer Cyclical	81
Fidelity Select Health Care Services Portfolio	Healthcare	1
Delaware Healthcare Fund;A	Healthcare	2
Kinetics Medical Fund;Advisor A	Healthcare	4
Fidelity Advisor Health Care Fund;A	Healthcare	6
PGIM Jennison Health Sciences Fund	Healthcare	9
Putnam Global Health Care Fund	Healthcare	12
DWS Health and Wellness Fund	Healthcare	16
Eaton Vance Worldwide Health Sciences Fund	Healthcare	20
Fidelity Select Medical Technology and Devices Ptf	Healthcare	21
Perkins Discovery Fund	Healthcare	24
Eventide Healthcare & Life Sciences Fund	Healthcare	27
Janus Henderson Global Life Sciences Fund	Healthcare	28
Fidelity Advisor Biotechnology Mutual Fund Class A	Healthcare	31
BlackRock Health Sciences Opportunities Port	Healthcare	39
Alger Health Sciences Fund	Healthcare	41
Rydex Health Care Fund	Healthcare	43
Invesco Health Care Fund	Healthcare	44
Fidelity Select Pharmaceuticals Portfolio	Healthcare	46
Hartford Healthcare Fund	Healthcare	47
Vanguard Health Care Fund	Healthcare	48
Vanguard Health Care Index Fund	Healthcare	49
T Rowe Price Health Sciences Fund	Healthcare	50
Virtus AllianzGI Health Sciences Fund	Healthcare	51
Schwab Health Care Fund	Healthcare	62
Franklin Biotechnology Discovery Fund	Healthcare	64
Fidelity Select Biotechnology Portfolio	Healthcare	65
Live Oak Health Sciences Fund	Healthcare	68
Rydex Biotechnology Fund	Healthcare	69
Hartford Healthcare HLS Fund	Healthcare	72
Saratoga Health & Biotechnology Portfoli	Healthcare	74
Upright Growth Fund	Tecnhonology	3
T Rowe Price Global Technology Fund	Tecnhonology	5
Janus Henderson Gl Tch and Innov Fd	Tecnhonology	7
Nationwide NYSE Arca Tech 100 Idx Fund	Tecnhonology	8
BNY Mellon Technology Growth Fund	Tecnhonology	10
Victory RS Science and Technology Fund	Tecnhonology	11
Jacob Internet Fund	Tecnhonology	13
MFS Technology Fund	Tecnhonology	14
Columbia Seligman Communications & Info Fd	Tecnhonology	15
Ivy Science & Technology Mutual Fund	Tecnhonology	17
USAA Science & Technology Fund	Tecnhonology	19
Goldman Sachs Technology Opportunities	Tecnhonology	22
BlackRock Technology Opportunities Fund	Tecnhonology	23
Fidelity Select Computers Portfolio	Tecnhonology	25
Fidelity Select Software and IT Svcs Ptf	Tecnhonology	26
Fidelity Select Technology Portfolio	Tecnhonology	29
Virtus AllianzGI Technology Fund	Tecnhonology	30
Fidelity Select IT Services Portfolio	Tecnhonology	32
Fidelity Select Communications Equipment Portfolio	Tecnhonology	33
Fidelity Select Semiconductors Portfolio	Tecnhonology	34
Rydex Electronics Fund	Tecnhonology	35
Columbia Global Technology Growth Fund	Tecnhonology	36
Invesco Technology Mutual Fund Class A	Tecnhonology	37
Fidelity Advisor Semiconductors Fund	Tecnhonology	38
Firsthand Alternative Energy Fund	Tecnhonology	40
Rydex Technology Fund	Tecnhonology	42
DWS Science and Technology Fund	Tecnhonology	45
Berkshire Focus Fund	Tecnhonology	52
ICON Health and Information Technology Mutual Fund Class Institutional	Tecnhonology	53
Wireless Fund	Tecnhonology	54
Firsthand Technology Opportunities Fund	Tecnhonology	55
T Rowe Price Science & Technology Fund	Tecnhonology	56
Columbia Seligman Global Technology Fund	Tecnhonology	57
Saratoga Technology & Communications Portfolio	Tecnhonology	58
Nationwide Bailard Technology & Science Fund	Tecnhonology	59
Fidelity Advisor Communications Equipment Mutual Fund Class A	Tecnhonology	60
Hennessy Technology Fund Investor	Tecnhonology	61
Janus Henderson Global Technology and Innovation Mutual Fund Class Institutional	Tecnhonology	63
Black Oak Emerging Technology Fund	Tecnhonology	66
Putnam Global Technology Mutual Fund Class A	Tecnhonology	70
Red Oak Technology Select Fund	Tecnhonology	71
VALIC Company I Science and Technology Mutual Fund	Tecnhonology	73
Rydex Internet Fund	Tecnhonology	75
Fidelity Advisor Technology Fund	Tecnhonology	76

**Table 12:** Efficiency scores for mutual funds under study for 1 year

Fund	Sector	${d}^{*}$	${w}_{VAR}^{*}$	${w}_{SD}^{*}$	${w}_{BETA}^{*}$	${w}_{RSQ}^{*}$	${w}_{SHARPE}^{*}$	${w}_{ALPHA}^{*}$
78	Consumer Cyclical	**− 0.127**	0.936	0.841	0.927	0.770	0.893	0.807
77	Consumer Cyclical	0.018	0.946	0.860	0.872	0.329	0.724	0.631
81	Consumer Cyclical	0.019	0.946	0.860	0.875	0.349	0.724	0.637
79	Consumer Cyclical	0.043	0.925	0.822	0.838	0.326	0.679	0.612
18	Consumer Cyclical	0.097	0.881	0.753	0.749	0.322	0.561	0.464
80	Consumer Cyclical	0.136	0.813	0.664	0.703	0.410	0.675	0.665
67	Consumer Cyclical	0.140	0.837	0.694	0.745	0.513	0.456	0.441
6	Healthcare	**− 0.048**	0.997	0.980	0.932	0.363	0.546	0.560
21	Healthcare	**− 0.038**	0.974	0.919	0.933	0.698	0.686	0.767
31	Healthcare	**− 0.025**	0.918	0.810	0.948	0.978	0.199	0.558
64	Healthcare	0.001	0.919	0.812	0.939	0.965	0.160	0.515
46	Healthcare	0.006	0.986	0.949	0.902	0.432	0.127	0.337
41	Healthcare	0.007	0.960	0.889	0.907	0.722	0.433	0.627
28	Healthcare	0.008	0.971	0.912	0.908	0.619	0.381	0.545
16	Healthcare	0.011	0.986	0.949	0.867	0.204	0.269	0.388
50	Healthcare	0.013	0.976	0.923	0.887	0.459	0.555	0.623
2	Healthcare	0.014	0.969	0.907	0.886	0.557	0.133	0.356
20	Healthcare	0.014	0.983	0.940	0.853	0.189	0.242	0.367
62	Healthcare	0.015	0.982	0.939	0.848	0.173	0.284	0.391
12	Healthcare	0.015	0.982	0.939	0.857	0.209	0.302	0.409
47	Healthcare	0.015	0.981	0.937	0.868	0.278	0.426	0.500
72	Healthcare	0.016	0.980	0.935	0.865	0.280	0.432	0.505
69	Healthcare	0.018	0.941	0.851	0.886	0.754	0.231	0.487
51	Healthcare	0.018	0.978	0.930	0.843	0.194	0.314	0.414
49	Healthcare	0.018	0.978	0.930	0.830	0.109	0.389	0.450
39	Healthcare	0.019	0.978	0.929	0.841	0.202	0.334	0.429
43	Healthcare	0.019	0.978	0.928	0.842	0.196	0.417	0.483
48	Healthcare	0.022	0.975	0.922	0.828	0.181	0.168	0.311
44	Healthcare	0.026	0.969	0.907	0.849	0.370	0.279	0.422
74	Healthcare	0.028	0.968	0.907	0.820	0.242	0.163	0.312
68	Healthcare	0.031	0.966	0.900	0.816	0.261	0.212	0.350
1	Healthcare	0.033	0.958	0.884	0.832	0.418	0.430	0.550
9	Healthcare	0.037	0.929	0.829	0.851	0.700	0.444	0.670
27	Healthcare	0.038	0.816	0.668	0.911	0.890	0.209	0.306
4	Healthcare	0.047	0.941	0.850	0.794	0.447	0.020	0.213
65	Healthcare	0.063	0.890	0.766	0.879	0.589	0.243	0.158
24	Healthcare	0.211	0.654	0.501	0.709	0.446	0.582	0.608
13	Tecnhonology	**− 0.118**	0.668	0.513	0.721	0.593	0.798	0.947
40	Tecnhonology	**− 0.043**	0.823	0.677	0.982	0.235	0.634	0.601
25	Tecnhonology	**− 0.020**	0.935	0.840	0.919	0.372	0.848	0.836
56	Tecnhonology	**− 0.002**	0.949	0.867	0.882	0.211	0.834	0.672
34	Tecnhonology	**− 0.002**	0.929	0.829	0.934	0.634	0.779	0.795
38	Tecnhonology	0.001	0.930	0.831	0.934	0.629	0.772	0.786
35	Tecnhonology	0.028	0.907	0.793	0.903	0.600	0.731	0.772
8	Tecnhonology	0.033	0.957	0.881	0.842	0.173	0.559	0.404
7	Tecnhonology	0.040	0.935	0.839	0.846	0.161	0.636	0.505
63	Tecnhonology	0.045	0.931	0.832	0.839	0.161	0.627	0.498
71	Tecnhonology	0.047	0.949	0.866	0.839	0.179	0.488	0.463
66	Tecnhonology	0.050	0.913	0.803	0.870	0.420	0.697	0.661
37	Tecnhonology	0.053	0.934	0.837	0.845	0.175	0.554	0.436
42	Tecnhonology	0.054	0.922	0.816	0.841	0.240	0.618	0.519
58	Tecnhonology	0.055	0.941	0.851	0.857	0.184	0.433	0.332
57	Tecnhonology	0.059	0.898	0.779	0.860	0.459	0.719	0.715
36	Tecnhonology	0.059	0.921	0.815	0.824	0.158	0.586	0.462
73	Tecnhonology	0.060	0.915	0.805	0.829	0.235	0.616	0.519
15	Tecnhonology	0.061	0.896	0.776	0.860	0.469	0.713	0.715
53	Tecnhonology	0.066	0.915	0.805	0.868	0.236	0.539	0.542
61	Tecnhonology	0.067	0.907	0.792	0.797	0.341	0.621	0.525
17	Tecnhonology	0.068	0.921	0.815	0.831	0.176	0.524	0.409
54	Tecnhonology	0.070	0.927	0.825	0.858	0.192	0.478	0.443
59	Tecnhonology	0.070	0.911	0.798	0.813	0.189	0.570	0.459
22	Tecnhonology	0.072	0.924	0.820	0.831	0.190	0.477	0.369
45	Tecnhonology	0.073	0.917	0.809	0.854	0.152	0.510	0.488
75	Tecnhonology	0.078	0.905	0.789	0.831	0.340	0.550	0.497
14	Tecnhonology	0.079	0.918	0.810	0.821	0.188	0.464	0.354
5	Tecnhonology	0.079	0.888	0.763	0.814	0.349	0.647	0.611
11	Tecnhonology	0.083	0.867	0.733	0.857	0.627	0.515	0.607
29	Tecnhonology	0.086	0.891	0.768	0.825	0.147	0.576	0.564
70	Tecnhonology	0.089	0.883	0.755	0.803	0.325	0.611	0.564
19	Tecnhonology	0.093	0.884	0.757	0.841	0.490	0.522	0.537
60	Tecnhonology	0.095	0.886	0.760	0.778	0.503	0.281	0.038
33	Tecnhonology	0.095	0.886	0.760	0.776	0.495	0.291	0.043
26	Tecnhonology	0.101	0.896	0.776	0.827	0.152	0.413	0.392
23	Tecnhonology	0.106	0.867	0.733	0.799	0.392	0.585	0.571
30	Tecnhonology	0.115	0.869	0.736	0.779	0.302	0.506	0.439
10	Tecnhonology	0.125	0.857	0.720	0.764	0.275	0.513	0.436
76	Tecnhonology	0.136	0.851	0.712	0.775	0.129	0.466	0.445
55	Tecnhonology	0.160	0.711	0.555	0.771	0.584	0.466	0.627
32	Tecnhonology	0.169	0.820	0.673	0.652	0.295	0.263	0.091
52	Tecnhonology	0.215	0.763	0.608	0.675	0.408	0.395	0.334
3	Tecnhonology	0.251	0.094	0.107	0.106	0.585	0.423	0.278

Bold means the efficient funds in each period

**Table 13:** Efficiency scores for mutual funds under study for 3 years

Fund	Sector	${d}^{*}$	${w}_{VAR}^{*}$	${w}_{SD}^{*}$	${w}_{BETA}^{*}$	${w}_{RSQ}^{*}$	${w}_{SHARPE}^{*}$	${w}_{ALPHA}^{*}$
78	Consumer Cyclical	**− 0.020**	0.807	0.699	0.757	0.418	0.574	0.534
79	Consumer Cyclical	0.038	0.843	0.742	0.725	0.247	0.631	0.339
77	Consumer Cyclical	0.072	0.841	0.740	0.712	0.221	0.511	0.241
81	Consumer Cyclical	0.075	0.839	0.737	0.710	0.226	0.503	0.235
80	Consumer Cyclical	0.156	0.689	0.574	0.501	0.443	0.449	0.241
67	Consumer Cyclical	0.177	0.722	0.607	0.523	0.456	0.225	0.022
18	Consumer Cyclical	0.187	0.772	0.660	0.535	0.297	0.251	0.026
27	Healthcare	**− 0.154**	0.596	0.489	0.808	0.917	0.318	0.448
21	Healthcare	**− 0.059**	0.925	0.852	0.690	0.469	0.779	0.415
4	Healthcare	**− 0.035**	0.984	0.948	0.812	0.468	0.292	0.116
65	Healthcare	**− 0.023**	0.776	0.664	0.860	0.713	0.281	0.341
41	Healthcare	**− 0.022**	0.896	0.812	0.685	0.642	0.472	0.247
46	Healthcare	**− 0.016**	0.977	0.937	0.782	0.388	0.552	0.245
20	Healthcare	**− 0.013**	0.989	0.958	0.772	0.209	0.585	0.239
31	Healthcare	**− 0.007**	0.848	0.748	0.635	0.706	0.307	0.135
39	Healthcare	**− 0.002**	0.973	0.930	0.737	0.224	0.639	0.273
12	Healthcare	0.001	0.985	0.951	0.771	0.263	0.546	0.225
64	Healthcare	0.002	0.794	0.684	0.575	0.747	0.185	0.032
62	Healthcare	0.005	0.984	0.949	0.755	0.194	0.439	0.159
51	Healthcare	0.011	0.973	0.930	0.736	0.215	0.585	0.241
16	Healthcare	0.012	0.976	0.935	0.739	0.205	0.458	0.170
68	Healthcare	0.016	0.970	0.924	0.745	0.333	0.280	0.084
74	Healthcare	0.017	0.969	0.923	0.740	0.315	0.171	0.019
48	Healthcare	0.021	0.967	0.919	0.721	0.230	0.407	0.142
28	Healthcare	0.022	0.944	0.882	0.714	0.406	0.520	0.233
1	Healthcare	0.025	0.884	0.795	0.647	0.579	0.456	0.224
49	Healthcare	0.028	0.960	0.908	0.692	0.159	0.542	0.210
6	Healthcare	0.038	0.934	0.866	0.678	0.361	0.513	0.223
44	Healthcare	0.043	0.938	0.872	0.675	0.300	0.471	0.187
72	Healthcare	0.045	0.929	0.858	0.659	0.327	0.506	0.213
2	Healthcare	0.047	0.917	0.840	0.672	0.460	0.280	0.084
50	Healthcare	0.047	0.908	0.828	0.636	0.386	0.553	0.255
47	Healthcare	0.047	0.929	0.859	0.659	0.324	0.491	0.203
69	Healthcare	0.048	0.823	0.717	0.573	0.629	0.216	0.039
43	Healthcare	0.049	0.936	0.870	0.658	0.253	0.432	0.155
9	Healthcare	0.064	0.836	0.734	0.571	0.571	0.379	0.162
24	Healthcare	0.114	0.536	0.437	0.618	0.448	0.529	0.535
13	Tecnhonology	**− 0.393**	0.515	0.420	0.652	0.576	0.763	0.928
40	Tecnhonology	**− 0.116**	0.696	0.581	0.960	0.411	0.485	0.161
23	Tecnhonology	**− 0.044**	0.822	0.717	0.670	0.346	0.892	0.438
25	Tecnhonology	**− 0.015**	0.879	0.787	0.771	0.448	0.736	0.288
3	Tecnhonology	**− 0.011**	0.100	0.125	0.124	0.890	0.264	0.512
7	Tecnhonology	**− 0.006**	0.895	0.809	0.733	0.207	0.863	0.360
33	Tecnhonology	**− 0.005**	0.873	0.780	0.887	0.635	0.054	0.122
26	Tecnhonology	**− 0.003**	0.895	0.809	0.758	0.265	0.798	0.289
22	Tecnhonology	**0.000**	0.905	0.824	0.755	0.220	0.783	0.302
54	Tecnhonology	0.001	0.913	0.835	0.755	0.262	0.764	0.227
60	Tecnhonology	0.001	0.873	0.779	0.885	0.633	0.021	0.096
70	Tecnhonology	0.001	0.845	0.744	0.701	0.369	0.840	0.394
63	Tecnhonology	0.004	0.890	0.803	0.725	0.206	0.859	0.358
55	Tecnhonology	0.006	0.696	0.581	0.670	0.637	0.620	0.372
52	Tecnhonology	0.024	0.717	0.602	0.640	0.529	0.702	0.475
45	Tecnhonology	0.029	0.881	0.790	0.722	0.195	0.756	0.246
71	Tecnhonology	0.031	0.919	0.843	0.704	0.254	0.609	0.302
35	Tecnhonology	0.032	0.749	0.635	0.627	0.512	0.695	0.331
58	Tecnhonology	0.035	0.908	0.828	0.754	0.219	0.572	0.157
36	Tecnhonology	0.035	0.880	0.789	0.708	0.202	0.757	0.287
8	Tecnhonology	0.041	0.907	0.826	0.729	0.254	0.539	0.264
14	Tecnhonology	0.042	0.883	0.794	0.725	0.249	0.665	0.228
37	Tecnhonology	0.043	0.886	0.797	0.721	0.211	0.674	0.227
5	Tecnhonology	0.048	0.804	0.696	0.674	0.456	0.682	0.296
56	Tecnhonology	0.059	0.849	0.749	0.690	0.317	0.688	0.262
42	Tecnhonology	0.060	0.858	0.760	0.683	0.224	0.710	0.258
34	Tecnhonology	0.063	0.720	0.605	0.593	0.524	0.609	0.257
29	Tecnhonology	0.065	0.839	0.737	0.666	0.208	0.759	0.255
17	Tecnhonology	0.067	0.855	0.757	0.685	0.239	0.672	0.232
73	Tecnhonology	0.067	0.845	0.744	0.671	0.250	0.712	0.267
38	Tecnhonology	0.068	0.721	0.606	0.595	0.526	0.594	0.243
57	Tecnhonology	0.071	0.795	0.685	0.635	0.360	0.728	0.313
15	Tecnhonology	0.072	0.795	0.686	0.635	0.355	0.729	0.313
59	Tecnhonology	0.075	0.853	0.755	0.670	0.201	0.674	0.226
30	Tecnhonology	0.075	0.814	0.707	0.667	0.386	0.669	0.266
32	Tecnhonology	0.085	0.824	0.720	0.641	0.423	0.532	0.282
76	Tecnhonology	0.085	0.824	0.719	0.645	0.197	0.724	0.225
53	Tecnhonology	0.096	0.841	0.740	0.677	0.253	0.558	0.105
19	Tecnhonology	0.101	0.799	0.690	0.643	0.364	0.617	0.218
10	Tecnhonology	0.105	0.811	0.704	0.644	0.309	0.610	0.199
11	Tecnhonology	0.105	0.738	0.624	0.602	0.459	0.579	0.209
75	Tecnhonology	0.106	0.826	0.722	0.665	0.304	0.540	0.141
66	Tecnhonology	0.115	0.807	0.699	0.638	0.328	0.561	0.161
61	Tecnhonology	0.136	0.790	0.680	0.585	0.324	0.481	0.240

Bold means the efficient funds in each period

**Table 14:** Efficiency scores for mutual funds under study for 5 years

Fund	Sector	${d}^{*}$	${w}_{VAR}^{*}$	${w}_{SD}^{*}$	${w}_{BETA}^{*}$	${w}_{RSQ}^{*}$	${w}_{SHARPE}^{*}$	${w}_{ALPHA}^{*}$
79	Consumer Cyclical	0.046	0.901	0.813	0.644	0.193	0.612	0.557
78	Consumer Cyclical	0.053	0.867	0.768	0.664	0.345	0.451	0.587
77	Consumer Cyclical	0.061	0.902	0.816	0.631	0.164	0.500	0.445
81	Consumer Cyclical	0.063	0.901	0.814	0.630	0.167	0.491	0.438
18	Consumer Cyclical	0.101	0.869	0.770	0.445	0.245	0.309	0.241
67	Consumer Cyclical	0.104	0.831	0.722	0.433	0.380	0.353	0.315
80	Consumer Cyclical	0.123	0.792	0.676	0.383	0.380	0.386	0.348
27	Healthcare	**− 0.085**	0.673	0.553	0.721	0.742	0.411	0.700
21	Healthcare	**− 0.021**	0.937	0.867	0.623	0.425	0.673	0.659
4	Healthcare	**− 0.013**	0.982	0.945	0.726	0.363	0.206	0.280
62	Healthcare	**− 0.007**	0.990	0.962	0.683	0.152	0.405	0.386
39	Healthcare	**− 0.004**	0.979	0.941	0.650	0.163	0.540	0.483
65	Healthcare	**− 0.002**	0.771	0.653	0.767	0.675	0.290	0.500
46	Healthcare	**− 0.001**	0.978	0.939	0.707	0.336	0.317	0.352
12	Healthcare	**− 0.001**	0.987	0.955	0.685	0.195	0.404	0.392
41	Healthcare	0.002	0.897	0.808	0.558	0.495	0.560	0.598
20	Healthcare	0.005	0.984	0.949	0.672	0.196	0.364	0.363
68	Healthcare	0.010	0.976	0.934	0.662	0.260	0.246	0.285
51	Healthcare	0.011	0.974	0.931	0.635	0.171	0.441	0.413
49	Healthcare	0.011	0.972	0.926	0.607	0.118	0.486	0.437
16	Healthcare	0.012	0.977	0.937	0.639	0.158	0.373	0.362
48	Healthcare	0.013	0.975	0.932	0.646	0.204	0.338	0.343
74	Healthcare	0.013	0.973	0.929	0.650	0.250	0.162	0.219
2	Healthcare	0.018	0.947	0.885	0.618	0.352	0.392	0.406
28	Healthcare	0.020	0.951	0.891	0.608	0.296	0.441	0.434
6	Healthcare	0.025	0.945	0.880	0.577	0.267	0.479	0.460
1	Healthcare	0.026	0.908	0.824	0.566	0.456	0.444	0.476
64	Healthcare	0.029	0.808	0.694	0.444	0.597	0.222	0.275
72	Healthcare	0.029	0.945	0.881	0.565	0.241	0.453	0.433
47	Healthcare	0.029	0.946	0.882	0.566	0.239	0.438	0.421
44	Healthcare	0.031	0.950	0.889	0.583	0.241	0.340	0.342
43	Healthcare	0.032	0.951	0.891	0.563	0.187	0.391	0.374
50	Healthcare	0.037	0.929	0.856	0.536	0.270	0.497	0.478
31	Healthcare	0.038	0.820	0.709	0.452	0.571	0.294	0.351
9	Healthcare	0.076	0.856	0.753	0.428	0.418	0.386	0.410
69	Healthcare	0.087	0.828	0.718	0.409	0.478	0.254	0.279
24	Healthcare	0.104	0.689	0.568	0.556	0.435	0.506	0.651
3	Tecnhonology	**− 0.225**	0.099	0.122	0.127	0.967	0.006	0.039
13	Tecnhonology	**− 0.188**	0.688	0.567	0.623	0.537	0.644	0.948
40	Tecnhonology	**− 0.103**	0.815	0.703	0.955	0.365	0.425	0.536
55	Tecnhonology	**− 0.034**	0.806	0.692	0.606	0.512	0.742	0.755
71	Tecnhonology	**− 0.031**	0.952	0.892	0.636	0.288	0.775	0.692
23	Tecnhonology	**− 0.019**	0.882	0.787	0.574	0.283	0.896	0.701
25	Tecnhonology	**− 0.017**	0.922	0.845	0.702	0.340	0.783	0.539
70	Tecnhonology	**− 0.009**	0.903	0.816	0.619	0.296	0.880	0.677
33	Tecnhonology	**− 0.009**	0.923	0.845	0.853	0.561	0.332	0.495
7	Tecnhonology	**− 0.006**	0.934	0.862	0.651	0.197	0.904	0.650
54	Tecnhonology	**− 0.002**	0.949	0.887	0.695	0.226	0.679	0.414
63	Tecnhonology	**− 0.001**	0.930	0.857	0.642	0.196	0.905	0.652
22	Tecnhonology	0.000	0.938	0.869	0.674	0.221	0.805	0.569
60	Tecnhonology	0.000	0.922	0.845	0.851	0.560	0.305	0.471
26	Tecnhonology	0.003	0.930	0.857	0.678	0.205	0.807	0.515
58	Tecnhonology	0.007	0.946	0.882	0.676	0.186	0.676	0.451
8	Tecnhonology	0.012	0.947	0.884	0.647	0.234	0.659	0.566
52	Tecnhonology	0.013	0.798	0.683	0.548	0.474	0.715	0.760
45	Tecnhonology	0.019	0.925	0.849	0.644	0.147	0.782	0.476
36	Tecnhonology	0.019	0.922	0.845	0.620	0.188	0.829	0.589
14	Tecnhonology	0.023	0.923	0.847	0.640	0.232	0.740	0.521
56	Tecnhonology	0.031	0.911	0.828	0.619	0.264	0.757	0.551
37	Tecnhonology	0.033	0.922	0.844	0.621	0.191	0.701	0.476
42	Tecnhonology	0.038	0.913	0.832	0.596	0.178	0.737	0.506
34	Tecnhonology	0.043	0.810	0.697	0.496	0.429	0.687	0.566
30	Tecnhonology	0.043	0.874	0.776	0.567	0.312	0.745	0.565
35	Tecnhonology	0.043	0.823	0.712	0.521	0.420	0.678	0.548
59	Tecnhonology	0.044	0.907	0.823	0.576	0.165	0.735	0.502
38	Tecnhonology	0.045	0.811	0.698	0.498	0.429	0.675	0.552
73	Tecnhonology	0.046	0.902	0.815	0.582	0.210	0.757	0.536
5	Tecnhonology	0.049	0.860	0.758	0.561	0.358	0.686	0.525
32	Tecnhonology	0.049	0.893	0.803	0.553	0.352	0.600	0.552
29	Tecnhonology	0.049	0.891	0.799	0.571	0.162	0.812	0.516
11	Tecnhonology	0.052	0.829	0.719	0.508	0.372	0.700	0.552
17	Tecnhonology	0.054	0.906	0.821	0.588	0.200	0.651	0.434
57	Tecnhonology	0.055	0.866	0.766	0.544	0.305	0.719	0.539
15	Tecnhonology	0.058	0.866	0.766	0.542	0.300	0.712	0.531
53	Tecnhonology	0.059	0.900	0.812	0.603	0.202	0.601	0.327
76	Tecnhonology	0.061	0.881	0.785	0.550	0.155	0.788	0.491
75	Tecnhonology	0.062	0.893	0.802	0.580	0.243	0.636	0.434
66	Tecnhonology	0.070	0.882	0.787	0.558	0.272	0.585	0.392
19	Tecnhonology	0.074	0.875	0.777	0.550	0.274	0.617	0.424
61	Tecnhonology	0.074	0.877	0.781	0.513	0.306	0.499	0.455
10	Tecnhonology	0.078	0.871	0.773	0.534	0.247	0.651	0.450

Bold means the efficient funds in each period

## Footnotes

1 The population, existing in the database consulted, consists of 53 technological funds, 9 consumer funds and 37 health funds. Since there are funds with no data or with many flaws, we used a sample with complete data.
